# Transcription‐Related Dynamics from Immune Disability into Endogenous Innovation

**DOI:** 10.1002/advs.201900767

**Published:** 2019-09-30

**Authors:** Yanna Zhang, Qian Li, Panyan Hou, Yanan Lu, Huanhuan Yang, Xiaojuan Lin, Chao Su, Yuquan Wei, Xiulin Yang, Hanshuo Yang, Xia Zhao, Xiancheng Chen

**Affiliations:** ^1^ State Key Laboratory of Biotherapy/Collaborative Innovation Center for Biotherapy West China Hospital Sichuan University Keyuan Road 4 No. 1, High Technological Development Zone Chengdu Sichuan 610041 P. R. China; ^2^ Department of Gynecology & Obstetrics West China Hospital/Second Hospital Sichuan University No 20, Section 3, South Renmin Road Chengdu Sichuan 610041 P. R. China

**Keywords:** involution‐innovation, molecular microenvironment, rhythm dynamics, tridimensional biologics

## Abstract

So far, thymus involution in adults is believed to be irreversible, and endogenous innovation for thymus‐related immunodeficiency remains to be an intractable puzzle. With the expectation of addressing this dilemma, human ovarian surface epithelium (OSE) has been reengineered as epithelial‐mesenchymal transition (EMT)‐tridimensional‐spheroid biologics (ETSB) using a dynamic EMT‐3D‐floating system along with 160 Gy X‐ray‐amelioration, which inoculates subcutaneously into aging rhesus and athymic Balb/c*^nu/nu^* mice. Herein, it is bioinformatically validated that ETSB can reset Clock/Arntl‐Per3/Tim molecule rhythm dynamics to re‐prime thymus residual (parathyroid or fatty‐like invalid vesicles yet no thymic architecture) to evolutionary transcription with overall cortex‐medulla endogenized by TECs undergoing MET/EMT reversion. Rhythm dynamics immediately resettles the bHLH‐LTβR‐NFκB‐RelA/B loop as a cascade to provoke the core immune microenvironment for multifunctional innovation of dynamic TCR orchestration, with harmonious naïve T‐subsets and TRECs renewals (*P* < 0.005). Subsequently, peripheral biological burden and tumor metastasis dynamics are addressed by innovative TCR‐defense/attack dynamics quickly (*P* < 0.005 vs Control), yet without autoimmune indication to hosts. Moreover, a functional blockade of core‐rhythm dynamics deeply impedes the endogenous innovation of invalid thymus residual. Thus this study may help pioneer a prospective strategy to innovate panoramic central‐peripheral immune microenvironments and defense dynamics for immune‐deficient/aging victims.

## Graphical abstract

Epithelial‐mesenchymal transition (EMT)‐tridimensional‐spheroid biologics (ETSB) could reset Clock/Arntl‐Per3/Tim molecule rhythm dynamics to reprime thymus residual (parathyroid or fatty‐like invalid vesicles yet no thymic architecture) to evolutionary transcription with overall cortex‐medulla endogenized by thymic epithelial cells (TECs) undergoing MET/EMT reversion. Rhythm dynamics immediately resettles bHLH‐LTβR‐NFκB‐RelA/B loop as cascade to provoke core immune microenvironment for multifunctional innovation of dynamic TCR orchestration, with harmonious naïve T‐subsets and T cell receptor excision circles (TRECs) renewals.

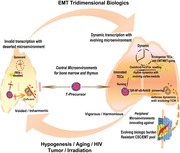

## 1 Introduction

Disharmony between overall systemic status and thymus‐related immune dysfunction plays a crucial role responsible for many immune‐deficient/aging disorders; where thymus‐related tissues/cells transplantation, despite having somewhat of an expectation to correct immune deficiency for humans, may cause organ‐specific autoimmune disorders or other immune issues due to potential HLA/MHC‐incompatibility of donor thymic tissues to recipient autologous systemic tissues. Essentially, only endogenous innovation could be used as a practicable and safe avenue to address immune disorders for humans, yet endogenous renovation for aging or disabled‐thymus remains a formidable biological puzzle.^1^, ^2^, ^3^ As the cradle and educators of immature T‐cells, thymus serves as dynamic role to mediate lymphopoietic activity. Yet thymus is the most rapidly aging tissue in normal individuals, with progressive involution toward quiescent rhythm beginning not later than puberty as disharmony of thymic evolutionary‐dynamics to host systemic level. Early thymus shares the same rudiment with parathyroid for development,^4^ with original roles in enhancing reproductive system and adolescence growth, which gives obvious evidence as an erstwhile endocrine organ.^5^, ^6^, ^7^ In fact, T‐cell production is not the original function of the thymus, but rather a recently evolved function. Thymic untimely involution may represent a disharmony for a still‐evolving erst endocrine organ to the biological burden of lymphopoietic activity. The “age‐related thymic involution” results in decreased output and functional capacity of T‐cell reservoir, and severely impairs the immune response to newly encountered antigens and increases incident of infection or tumor‐dependent morbidity and mortality, making thymic involution a major cause of the age‐related immune decline.^8^, ^9^ Thymus recession also impairs the capacity to recover adaptive immunity following immune depletion in patients.^10^, ^11^ Consequently, investigation to restore thymus function for aging hosts and, especially, the strategies aimed at dead‐end thymic rhythm renewal for immune deficient victims, would be beneficial in a wide variety of practical clinical settings including AIDS and tumor administration. As for thymic tissue architecture conversion, TECs would transition back and forth between epithelial and mesenchymal phenotypes (intrathymic epithelial‐mesenchymal transition (EMT)/MET) as resettling dynamic evolution or involution of thymus‐related bioclock to mediate organogenesis and senescence conversion.^12^, ^13^ Yet, owing to inharmonic involution of thymic bioactivity and architecture, peripheral T‐cell repertoire development could not match with EMT‐CSC constant evolution, resulting in significant increase in clinical cancer incidence and keeping CSC/EMT pool‐targeted strategy an intractable dilemma.^14^

Ovarian surface epithelium (OSE), like TECs, can transition back/forth between epithelial and mesenchymal phenotypes (EMT/MET) in normal or pathological settings such as follicular rupture and subsequent ovarian remodeling as well as in carcinogenesis.^15^ It is well known that infancy thymus in dynamic rhythm features superior cellular/humoral microenvironment as incubator for renewable T‐cell subsets. Once athymic or senile host in quiescent rhythm is addressed with rhythmic dynamics for central immunity resetting renewal microenvironments,^16^, ^17^ there would be the potential for thymic progenitors to be remodeled as evolutionary revival repertoire to adapt to biologic burden from EMT, CSC, evolutionary or targeted‐strategy‐resistant subsets.^18^ Recently, it has been proposed that 3D cell microenvironment could effectively enhance immunotherapy reactivity.^19^ In this study, 3D‐ameliorated biologics with rhythmic dynamics would be prepared from EMT/MET‐undergoing OSE and tried to see if retrogressive central‐peripheral defense axis in immune deficient/aging hosts could be innovated against evolutionary biological burden and refractory therapeutic targets.

## 2 Results

### 2.1 In Vitro EMT Model Establishment and EMT‐3D‐Spheroid Transcriptional Characteristics

**Figure 1:**
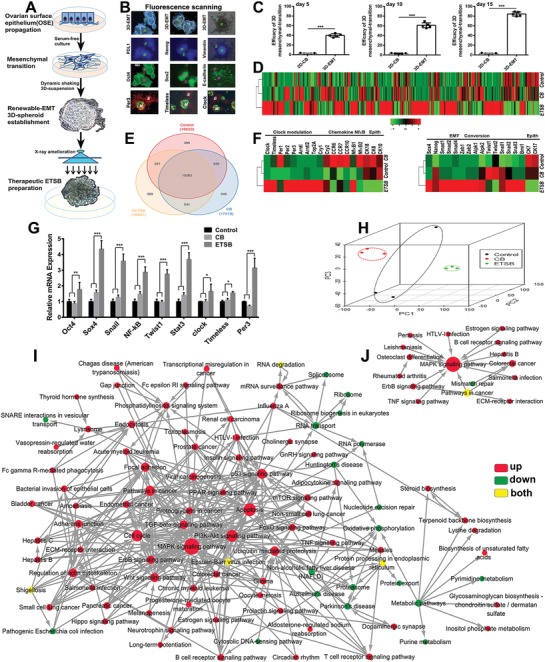
Dynamic establishment and bioinformatic characteristics of EMT‐3D‐spheroid conversion. A) Schematic depiction for therapeutic 3D‐ETSB establishment. B) Multiepitope phenotypes of EMT‐3D‐renewable spheroids cover Nanog, Oct‐4, Sox‐2, PDL‐1, Per3, Timeless, and Clock in mesenchymal‐transition parts but negative in nontransition parts (*M*: mesenchymal transition parts, Vimentin^+^; *E*: epithelial/non‐transition parts, E‐cadherin^+^). C) Dynamic comparison for the efficacy of mesenchymal‐transition between routine‐2D‐CB culture pattern and 3D‐floating‐EMT culture model. See also Figure S1A and Video S1 (Supporting Information). D) Whole transcriptome of 3D‐ETSB, 2D‐CB, and Control/OSE cells is showed as hierarchical clustering heat‐maps to see if the transcriptome of 3D‐ETSB is similar to that of 2D‐CB and Control/OSE cells or not. E) Three way Venn diagram based on whole transcriptome represents the distribution of the expressed genes among Control, CB, and ETSB. F) Gene expression of transcriptional factors and functional genes associated with Clock modulation and EMT conversion as indicated in ETSB, CB, and Control cells is showed using the left and right heat maps. G) qRT‐PCR analysis was adopted to evaluate the conversion dynamics of relevant TF in 3D‐ETSB versus 2D‐CB and Control (***P < 0.005). H) Whole transcriptomes were subjected to MDS on expressed genes to assess sample diversity and relatedness among 3D‐ETSB (green), 2D‐CB (red), and Control/wild OSEs (black). See also Video S3 (Supporting Information). I) Pathway‐Act‐Network analysis for ETSB versus Control. A node represents a signaling pathway. The node color is correlated with pathway expression pattern. Red indicates that the signaling pathway is activated, while green indicates that the signaling pathway is suppressed. Yellow indicates that the genes included in the corresponding signaling pathway are both upregulated and downregulated. Lines represent interactive relationship between signaling pathways. The direction of the arrow is from the cascade source to the target. J) As in (I), Pathway‐Act‐Network analysis for CB versus Control. See also Figure S1 and Videos S1 and S2, and Table S1 (Supporting Information).

3D‐ETSB are derived from human OSEs undergoing EMT/MET reversion (**Figure** **1**A). OSEs are propagated and unattached by dynamic suspension system to regenerate EMT‐3D‐spheroid pluripotent reversion in ameliorative DMEM/F12/1640‐integrated medium (Video S1, Supporting Information). More than 250 floating EMT‐3D‐spheroids per mL could be enriched for about 15 d of ameliorative dynamic suspension. Each 3D‐spheroid contains more than 320 EMT cells, with about 195 ± 25 µm/each D and developing positive‐phenotypes for Nanog, Oct‐4, Sox‐2, PDL‐1, Per3, Timeless and Clock in mesenchymal‐transition parts but negative in nontransition parts (Figure 1B). Routine 2D‐culture model could not generate 3D‐EMT spheroid (Figure 1C; Figure S1A, Supporting Information); EMT‐3D conversion was further verified by immunofluorescence dynamic scanning(Video S2, Supporting Information), with CD44^+^/CD133^+^for mesenchymal transition parts but negative in epithelial parts (Figure S1B, Supporting Information). FACS using CD44/CD73‐CD133/CD200 indicated about 85.5% of multiepitope expression index for EMT/MET dynamic transition cells (Figure S1C, Supporting Information). FPKM analyses display that transcriptome of 3D‐ETSB is not similar to that of 2D‐CB and Control/OSE cells (Figure 1D). Three way Venn diagram for whole transcriptome identifies the distribution of active genes among Control, CB, and ETSB (Figure 1E). For the expression patterns of active genes, it is found that ETSB has expressed transcriptional factors and functional genes associated with clock modulation and sequent EMT development, but losing epithelium differentiation (Figure 1F). Transition dynamics was also verified by qRT‐PCR, which revealed the elevating expression index for Oct‐4, Sox‐4, Snail, NF‐κB, Twist1 and Stat3, Clock, Tim, and Per3 (Figure 1G). Whole transcriptomes were subjected to multidimensional dynamic scaling to assess sample diversity and relatedness. ETSB symbols clustered more together, meaning closer relatedness, than CB. Meanwhile, ETSB is are very distinct from CB and Control groups, indicating diverse transcriptional characteristics among them and dynamic variation of wild OSEs/Control until transcription terminating (Figure 1H; Video S3, Supporting Information). Pathway‐Act‐Network analyses for ETSB versus Control (Figure 1I) and CB versus Control (Figure 1J) indicate the networks collectively modulating 3D‐EMT reversion by MAPK, Wnt, TCR, Cell cycle, PI3K‐Akt, and Circadian rhythm signals. ETSB:CB and ETSB:Control comparisons are displayed as Venn (Figure S1D, Supporting Information) and have recapitulated much more up‐/down‐regulated gene transcriptions than CB:Control comparison, corresponding to statistical analyses for DEG among groups (Figure S1E, Supporting Information). Transcriptome waveform distribution illustrates that ETSB/EMT‐3D‐spheroids have clustered higher expression density over 2D‐CB or Control (Figure S1F, Supporting Information, density refers to the ratio of the number of genes under the expression to total number of genes based on whole transcriptome RNAseq). Heat maps for TF‐DEG have revealed both higher expression and closer relatedness in ETSB than in 2D‐CB/Control (Figure S1G, Supporting Information). TF‐DEG interactive network analyses were adopted to monitor TF‐coding ability and identified upregulated transcriptions of Arntl/‐2, Clock and Per2/3 (bHLH‐TF) in both ETSB:Control and ETSB:CB comparisons yet not in CB:Control comparison (Figure S1H, Supporting Information). Clock/Arntl‐TF‐null OSEs could not generate EMT‐3D‐spheroid conversion, suggesting that 3D‐conversion may be involved in innate dynamic rhythm. In order to detect biodistribution in homeostatic animals, ETSB were subcutaneously injected into Balb/c*^nu/nu^* nude mice and imaged every other day (Figure S1I, Supporting Information), which indicated initial and eventual localization in subcutaneous tissues by day 10 and gradual diminishment until disappearance by about day 30. Thymus rudiments from the nude mice were nursed with 3D‐floating ETSB (no direct attachment) in ameliorative DMEM/F12/1640‐integrated medium for over four weeks, with consequent cortex‐medulla survived panoramically without toxicity reaction (Figure S1J, Supporting Information).

### 2.2 Transcription‐Related Dynamics of ETSB‐Reset Molecule Rhythm for Core Immunity

**Figure 2:**
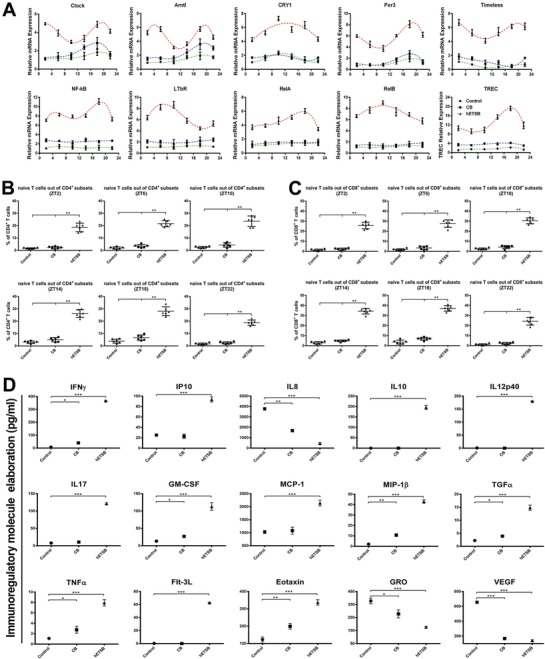
Transcription‐related dynamics of hETSB‐reset immune molecule rhythm. A) Dynamic oscillations in mRNA transcriptions of rhesus Clock/Arntl with other core genes during different zeitgeber times in respective groups were detected by qPCR assays. Data are presented as mean ± SD (*n* = 6). *P* < 0.05 for hETSB amplitude versus Control and CB at individual circadian time point (ZT2, ZT6, ZT10, ZT14, ZT18, and ZT22). B) Naïve TCR dynamic index via CD4:CD62L:CD45RA multiepitope expressions for rhesus T cell subsets during circadian zeitgeber times was detected 2 years after respective regimen termination by FACS assay. C) As in (B), Naïve TCR dynamic index via CD8:CD62L:CD45RA multiepitope expressions for rhesus T cell subsets during circadian zeitgeber times. D) As detected by magnetic bead microarray, multifunctional harmonious enhancement of immunoregulatory molecules covers IFN‐γ, IP‐10, IL‐10, IL‐12, IL‐17, GM‐CSF, MCP‐1/CCL2, MIP‐1β, TGFα, TNFα, Flt‐3L, and Eotaxin; comprehensive down‐regulation of molecules includes IL‐8, GRO/MGSA (Growth‐related oncogene) as well as selective VEGF depletion in ETSB group (*n* = 6). (Values expressed as means±SD at ZT2. *P < 0.05; ** *P* < 0.01; ***P < 0.005 versus control or CB groups). See also Figure S2 and Table S5 (Supporting Information).

**Figure 3:**
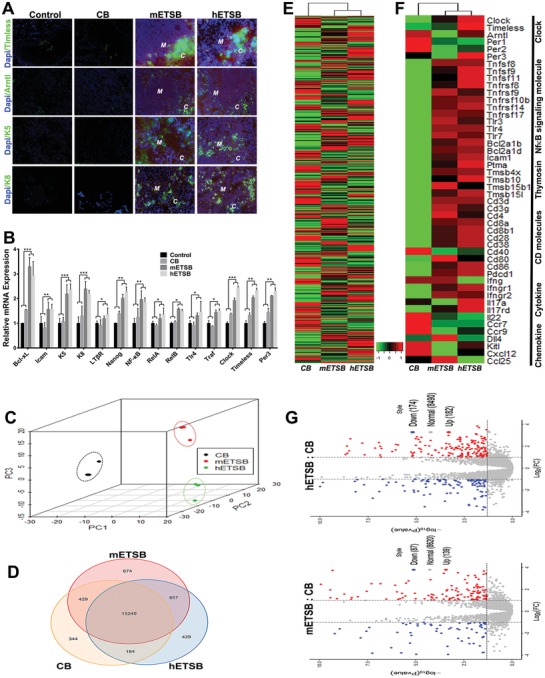
ETSB reprimes rhythm‐related central microenvironments into dynamic transcription. A) Core Arntl/Timeless molecules resetting TEC‐innovated hotspots (Panel 3–4, K8‐K5^+^ TEC‐endogenized subsets) could be detected in 13–14 week of Post‐Balb/c*^nu/nu^* by confocal dynamic scan, with renovated thymic cortex (*c*) and medulla (*m*) indicated and compared with CB/Control (*n* = 6). B) Transcriptional revival of Bcl‐xL, Icam, K5, K8, LTβR/(mTEC), Nanog, NF‐κB, RelA, RelB, Tlr4, Traf, Clock, timeless, and Per3 for TECs by qRT‐PCR in different groups was identified to monitor if the quiescent thymic rhythm was reset by bHLH‐Clock axis with MET/EMT‐undergoing TECs innovation favorable for ETSB‐primed core microenvironment revival from vesicular residual in voided microenvironment (**P < 0.01; ***P < 0.005). C) Transcriptomes for thymocytes (no thymopoiesis for Control) were obtained by RNA‐seq and subjected to MDS on expressed genes. Symbols of each transcriptome cluster together, indicating replicates closely related within each sample. D) Venn diagram based on whole transcriptome RNAseq for thymus illustrates overlapping distribution of gene expression among CB, mETSB, and hETSB groups (Control group without functional thymus). E) Expression profiles of active genes are shown as hierarchical clustering heat‐maps. Red represents elevated expression while green represents decreased expression, compared with the row mean. Each column represents an average value based on three biologic replicate. F) FPKM‐normalized identification of DEGs in thymocytes from three groups (Control with no functional thymus) was displayed as heatmaps to find what feedback regulatory cascades or TF‐families were involved crucially in tridimensional core microenvironment modulation. G) Volcano plots represent thymic DEGs between two group comparisons (mETSB:CB; hETSB:CB), with maximum adjustment in hETSB:CB. Red dots indicate upregulation in DEG (Log_2_FoldChange ≥ 1; FDR adjusted *P* value or padj ≤ 0.05), and blue dots indicate down regulation (Log_2_Foldchange ≤ ‐1; padj ≤ 0.05). See also Figure S3 (Supporting Information).

Aging rhesus macaques were subjected to five times of hETSB inoculations (Month 0‐1‐6‐12‐24 protocol) or other corresponding regimens during 24 months (Figure S2A, Supporting Information). Two years after ETSB inoculation procedure termination, animals of ETSB group appeared biologically younger than Control and CB groups (Figure S2B, Supporting Information). MRI scanning has identified endogenous revival of thymic lobes in front of trachea in elder animal of hETSB group, yet not in Control or CB groups (Figure S2C, Supporting Information). Thymic size and weight of hETSB group are multifold over CB and Control groups (Figure S2D, Supporting Information). Yet, lung and spleen weights keep similar among different groups (Figure S2E, Supporting Information). Transcriptional oscillations of core rhythm genes (Clock/Arntl/Cry1/Per3/Timeless) during circadian zeitgeber times have been evidently enhanced in hETSB group, with consequent fluctuations for LTβR‐NF‐κB family and TRECs versus Control and CB groups (**Figure** **2**A and Table S5, Supporting Information). CD4:CD62L:CD45RA and CD8:CD62L:CD45RA multiepitope expressions during circadian zeitgeber times for rhesus T cell subsets via FACS have further verified harmonious revival of naïve TCR dynamic index in hETSB group yet not in other groups (Figure 2B,C). As peripheral microenvironments detected by magnetic bead microarray, multifunctional enhancement of immunoregulatory molecules covers IFN‐γ, IP‐10, IL‐10, IL‐12, IL‐17, GM‐CSF, MCP‐1/CCL2, MIP‐1β, TGFα, TNFα, Flt‐3L, and Eotaxin; collective down‐regulation of molecules involves IL‐8, GRO/MGSA (Growth‐related oncogene) as well as selective VEGF depletion in hETSB group (Figure 2D). Moreover, Balb/c*^nu/nu^* nude mice 6–7 weeks of age were subjected to four times of ETSB inoculation or other corresponding regimens by week 0‐2‐3‐4 protocol, (Figure S2F, with primary inoculation followed by second inoculation 2 weeks later, then by one week apart). All hosts manifested athymic status before receiving corresponding inoculation regimens on thorax MRI. Posttherapy MRI scanning revealed endogenous revival of thymic lobes in front of trachea in m/hETSB groups yet not in Control/CB group(Figure S2G, Supporting Information). In situ detection verified thymic left/right lobes renovated as tridimensional heart‐size in ETSB groups with no newborn hair in skin. Panoramic histomorphometry illustrates the endogenized cortex‐medulla in ETSB groups as core microenvironments tridimensionally remodeled from vesicular rudiment; yet in CB group few lymphocyte‐like cells recruited around the low or not endogenized vesicular residual without cortex‐medulla renovation. Meanwhile, parathyroid‐like vesicles have fully retained in Control group as no lymphocyte‐like cells recruited around the voided microenvironments (Figure S2H, Supporting Information). Final thymic sizes in ETSB groups are multifold as 3D‐renovated over vesicular rudiment (Figure S2I, Supporting Information). qRT‐PCR for MHC/HLA of TECs revealed the enhanced MHC yet without HLA expression in hETSB group, thus excluding exogenous TEC transition and meaning involutional thymus rudiment reversed into innovation (Figure S2J, Supporting Information). Thymocytes/TECs ratio for the renovated cortex and medulla in ETSB groups is multifold over CB group (Figure S2K, Supporting Information). Confocal dynamic scan detects central Bmal1/Arntl‐Tim molecules resetting TEC‐innovated microenvironment in the renovated cortex and medulla (K8‐K5^+^ TEC in situ subsets) yet not in the vesicular rudiment (**Figure** **3**A). qRT‐PCR for TECs revealed the elevating transcriptional index of Bcl‐xL, Icam, K5, K8, LTβR, Nanog, NF‐κB, RelA, RelB, Tlr4, Traf, Clock, timeless and Per3 in m/hETSB groups as invalid thymic rhythm was reversed by bHLH‐Clock axis with MET/EMT‐undergoing favorable for microenvironment revival from the voided vesicular residual (Figure 3B). RNA‐seq for thymocytes was subjected to MDS, which reveals that symbols from m/hETSB groups share closer relatedness with each other yet only poor relatedness with CB symbols, meaning transcriptional characteristics of CB group deviant from m/hETSB groups (Figure 3C, Control group with no functional thymopoiesis). Venn diagram for whole transcriptome of thymus illustrates overlapping distribution of the active genes among CB, mETSB, and hETSB groups (Figure 3D). Global expression profiles of thymic genes are shown as heat maps, with distinct expression profiles in different groups yet close relatives of expression profiles between m‐ and h‐ ETSB groups, implicating systemic revival of central immunoregulatory axis (Figure 3E). FPKM‐normalized DEG from thymocytes of different groups was displayed on heat map (Figure 3F, Control without functional thymus). Upregulated DEGs mainly cover bHLH‐TF: Clock/Arntl‐Tim/Per3 and NF‐κB signals, Thymosin, CD antigens, and IFN families, indicating relevant gene transcription involved crucially in bHLH/Clock feedback loop with EMT/MET reversion for core microenvironment revival. Active genes in two group comparison (mETSB:CB; hETSB:CB) are displayed as Volcano plots (Figure 3G), with maximum adjustment in hETSB:CB comparison meaning evolutionary modification in thymic molecule microenvironment. Expression diversity in TECs of medulla and cortex were verified by thymic transcriptome and crucially involved in transcriptional factors and active genes associated with central immune microenvironment by Clock modulation and EMT conversion (Figure S3A, Supporting Information). Additionally, KEGG analysis for thymic transcriptome from ETSB groups versus CB group indicates that NF‐κB, Notch, Wnt, TNF, cell cycle, NK, Th1/Th2/Th17 differentiation, TCR and IL‐17 signals modulate core immune microenvironments collectively (Figure S3B, Supporting Information). Transcriptional innovation in Clock, timeless, Per3, Arntl, Bcl‐xL, Icam, RelA, RelB, CD3γ, IFNγ, and Pdcd1 for thymocytes in ETSB groups was identified by qRT‐PCR (Figure S3C, Supporting Information). The central‐phased in situ TCR/CD3 orchestrations were illustrated by FACS assay (Figure S3D, Supporting Information). Dynamic index of *αβ*/*γδ*TCR repertoire of various immune cells in remodeled central microenvironments is very complementary to one another for the periods from week 11 to week 15 (Figure S3E, Supporting Information).

### 2.3 Transcription‐Related Dynamics of ETSB‐Primed Molecule Microenvironment Innovation

**Figure 4:**
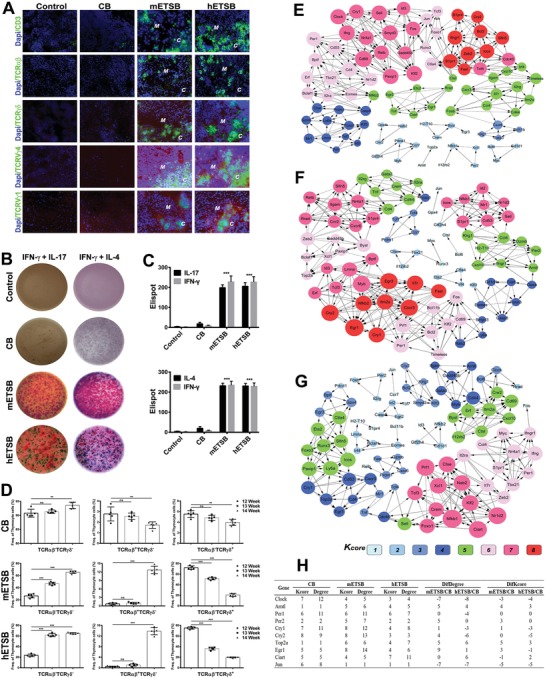
Transcription‐related dynamics of ETSB‐reset core immune microenvironment. A) Immune molecule confocal‐scanning assay was used to reveal multifunctional revival levels of naive TCR repertoire from thymic rudiment in 13–14 week of Post‐Balb/c*^nu/nu^* nude mice (***C***, in situ reoriginated thymus cortex; ***M***, in situ reoriginated medulla) compared with CB/Control. B) Multi/Dual‐color ELISpot assay was adopted to monitor the reactivity of endogenous thymic innovation versus peripheral biologic burden with collective expression of IFN‐γ (red spots) and IL‐17 (green spots)/IL‐4 (blue spots) from thymocytes detected by histomorphometry after the impact of ETSB cells as in vitro biological burden, yet with no functional thymocytes in Control verified by the assay (*n* = 5). C) As in (B), graph depicts the differences between ETSB and CB groups. *P < 0.05, **P < 0.01, ***P < 0.005. D) FACS assay for thymocytes illustrates central‐phased in situ *αβ*/*γδ*TCR dynamic index from week 12 to week 14 in ETSB and CB groups (*n* = 5, no thymopoiesis in Control). E) Coexpression networks are populated with k‐core algorithm according to the degree centrality and normalized signal intensity of dynamic rhythm‐related genes in CB group. The lines represent correlative relationships via solid line for positively corrected and dashed line for negatively corrected with direction from the cascade source to the target. F) As in (E), Coexpression networks with k‐core algorithm for mETSB group. G) As in (E), Coexpression networks were populated with k‐core algorithm for hETSB group. H) Intersections of relevant genes are listed by the difference k‐core scoring/degree among mETSB/CB and hETSB/CB groups. Consequently, Arntl/Clock Feedback regulatory networks are highly connected with dynamic modulation of rhythm‐related pathway for thymic endogenous innovation. See also Figure S4 (Supporting Information).

Central TCR repertoire revivals were further validated by in situ confocal‐scanning, which revealed periodical multifunctional TCR expression index for naive TCR renovating hotspots (**Figure** **4**A). Reactivity of endogenous thymic renovation versus peripheral biologic burden was identified by Multi‐color ELISpot assay, which demonstrated collective expression of IFN‐γ (red spots) and IL‐17(green spots)/IL‐4(blue spots) by thymocytes from ETSB groups, yet not from CB groups (Figure 4B). Histomorphometry algorithm detected no functional thymocyte from Control (Figure 4C). FACS assay illustrates central‐phased in situ TCR dynamic index (Figure 4D), with *αβ*/*γδ*TCR TCR orchestration index over 12%/20% at week 14 in m/hETSB groups yet under 2%/4% in CB group, without functional TCR development in Control. Coexpression networks (Figure 4E) populated with k‐core algorithm of relevant DEGs reveal the degree centrality and normalized signal intensity of dynamic rhythm‐related genes in CB, mETSB(Figure 4F), and hETSB groups (Figure 4G). Lines represent correlative relationships via solid line for positively corrected and dashed line for negatively corrected with direction from the source to the target. Volume of the node represents the degree centrality of coexpression; different colors represent the corresponding k‐core scoring. Intersections of relevant genes are listed by difference k‐core scoring and degree among mETSB:CB and hETSB:CB groups (Figure 4H). The greater the value of k‐core, the stronger DEGs coexpressed. Consequently, Arntl/Clock feedback regulatory network is highly connected with dynamic modulation of rhythm‐related pathway for thymic endogenous innovation.

As dynamic core‐peripheral immunoregulatory network scan, 200× confocal magnification illustrates distribution of various immune cells in renovated thymus, spleen and LN in ETSB‐inoculated athymic mice of week 14–15, which detects the evolutionary revival of core‐peripheral cell microenvironments (Figure S4A, Supporting Information). Global expression profiles are shown as heat maps. Different groups have distinct expression profiles; yet with close relatives between m/hETSB profiles(Figure S4B, Supporting Information), implicating systemic orchestration of central‐peripheral immune axis. As subjected to MDS on expressed genes, unorchestrated splenocytes from CB and Control groups cluster much closely together than multifunctional ETSB able to address peripheral multiple burdens (Figure S4C and Video S4, Supporting Information). Venn diagram for whole transcriptome of spleen represents the overlapping distribution of gene expressions among Control, CB, mETSB, and hETSB groups (Figure S4D, Supporting Information). Volcano plots of splenic transcriptome represent maximum adjustment in hETSB:Control versus mETSB:Control next CB:Control among two group comparisons (Figure S4E, Supporting Information), meaning evolutionary modification of peripheral molecule microenvironment. FPKM‐normalized DEG identification for splenocytes displays main gene families with evident upregulation covering bHLH‐TF (Clock‐Arntl/Per2), NF‐κB‐Traf‐Bcl2, Thymosin, CD3 and IL17‐IFN, with relative gene transcription involved crucially in bHLH‐NF‐κB cascade networks to peripheral microenvironment modulation in hETSB group (Figure S4F, Supporting Information), indicating systemic revival of central‐peripheral defense. qRT‐PCR further revealed the elevating transcription index for CD40, RelA, RelB, Icam, Tlr4, Traf, Bcl‐2, Bcl‐xL, NF‐κB and LTβR in spleen of ETSB groups (Figure S4G, Supporting Information). KEGG comparison for splenocytes from m/hETSB or CB groups versus Control group identifies that networks involved commonly in peripheral molecule microenvironment modification cover NF‐κB, Wnt, TCR, Th1/Th2/Th17 differentiations, NK‐cytotoxicity, CAMs and Leukocyte transendothelial signals (Figure S4H, Supporting Information).

### 2.4 Innovation of Core‐Immune Deficiency Requires Clock/Arntl‐Tim Dynamic Modulation

**Figure 5:**
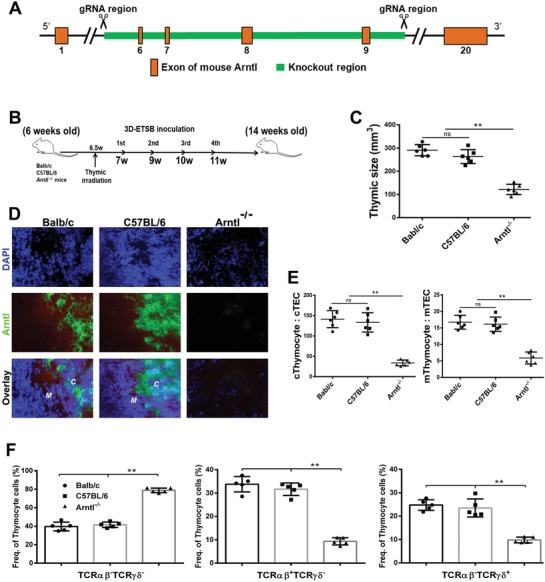
Innovation of core‐immune deficiency requires dynamic modulation of Clock/Arntl‐Tim loop. A) Schematic depiction for Arntl/Bmal1^−/−^ mice via deletion of exon 6,7,8 and 9 in arntl gene locus using CRISPR/Cas9 techniques. B) Relevant experimental layout in Arntl*^−/−^* hosts with normal Balb/c and C57BL/6 mice as control. C) Graph depicts the thymic sizes to see whether thymus‐irradiated residues would be 3D‐renovated among different groups. *P < 0.05, **P < 0.01. D) Confocal dynamic scanning assay was used to detect whether central Arntl/Bmal1 molecules reset in situ thymus after targeted thymic irradiation and 3D‐biologics administrations (***C***, renovated thymus cortex; ***M***, medulla; *n* = 6). E) Graphs represent the thymocytes/TECs ratio for medulla (m‐) and cortex (c‐) of three groups (**P < 0.01). F) FACS assay for thymocytes further illustrates the central‐phased *αβ*/*γδ* in situ naïve TCR index so as to compare TCR orchestration indications among different groups; *n* = 5; week 12–13; **P < 0.01. See also Figure S5 (Supporting Information).

*Arntl/Bmal1^−/−^* model was established via deleting exon 6,7,8 and 9 in arntl gene locus with CRISPR/Cas9 techniques (**Figure** **5**A). Targeted thymic irradiation for the knockout mice was performed at week 6–7 and then subjected to four times of 3D‐ETSB inoculation with normal Balb/c and C57BL/6 mice as control (Figure 5B). Thymus‐irradiated residues are 3D‐renovated in control Balb/c and C57BL/6 mice yet not in *Arntl/Bmal1^−/−^* mice (Figure 5C). Confocal dynamic scan has detected Arntl/Bmal1 molecules resetting in situ thymus in normal Balb/c and C57BL/6 mice as control, yet not in *Arntl/Bmal1^−/−^* mice (Figure 5D). ETSB could not renovate cortex/medulla of thymus for *Arntl/Bmal1^−/−^* mice. Thymocytes/TECs ratio for the renovated cortex and medulla in control Balb/c and C57BL/6 mice is multifold over *Arntl/Bmal1^−/−^* mice (Figure 5E). FACS assay for thymocytes illustrates that central‐phased *αβ*/*γδ* in situ naïve TCR development index has been evidently depressed in *Arntl/Bmal1^−/−^* mice (Figure 5F). Aging *Arntl/Bmal1^−/−^* hosts were subjected to four times of ETSB inoculation with aging Balb/c and C57BL/6 mice as control (Figure S5A, Supporting Information). Thymic sizes of aging hosts keep similar among different groups before ETSB administration (Figure S5B, Supporting Information). Yet, thymic sizes in aging Balb/c and C57BL/6 mice are multifold over aging *Arntl/Bmal1^−/−^* mice after ETSB administration (Figure S5C, Supporting Information). Central‐phased *αβ*/*γδ* TCR positive‐index keeps similar levels among different groups before ETSB administration (Figure S5D, Supporting Information). TCR‐positive index was evidently depressed in aging *Arntl/Bmal1^−/−^* mice than aging Balb/c or C57BL/6 mice after ETSB administration (Figure S5E, Supporting Information).

**Figure 6:**
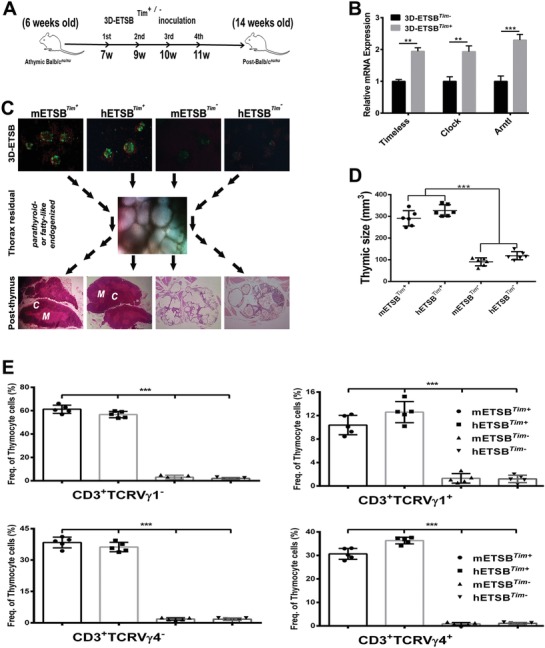
Clock/Arntl‐Tim dynamic blockade impedes thymic rudiment innovation. A) Relevant experimental layout of Clock/Arntl‐Tim‐low/negative ETSB (3D‐ETSB*^Tim−^*) on Balb/c*^nu/nu^* mice. B) Functional blockade of Clock/Arntl‐Tim feedback regulatory network in 3D‐biologics was evaluated using qRT‐PCR. C) Panoramic histomorphometry has monitored the different priming‐dynamics of homeostatic ETSB and ETSB*^Tim−^* to drive invalid fatty‐like or parathyroid vesicles into evolutionary innovation of cortex (*C*)/medulla (*M*) as functional parenchyma endogenized by TECs undergoing MET/EMT reversion from vesicular thymic residual with no cortex‐medulla. D) Thymic sizes of homeostatic ETSB and ETSB*^Tim−^* groups at week 14 (***P < 0.005; *n* = 6). E) FACS assay manifests TCR defense dynamics based on central *γδ*TCR/in situ naïve TCR index at week 12 between homeostatic ETSB and ETSB*^Tim−^* groups, *n* = 5. See also Figure S6 (Supporting Information).

Balb/c*^nu/nu^* nude mice were subjected to four times of homeostatic ETSB or ETSB*^Tim−^* inoculation (**Figure** **6**A). The blockade of Clock/Arntl‐Tim feedback regulatory network by Lipo‐Que modification at day 14 for 3D‐ETSB*^Tim−^* was further evaluated with qRT‐PCR analysis (Figure 6B). Unlike homeostatic ETSB, the ETSB*^Tim−^* has lost the activity to revivify parathyroid‐or fatty‐like epithelial rudiment into endogenous innovation of cortex (*c*) and medulla (*m*) as evolutionary thymic parenchyma (Figure 6C). Finally, thymic size in homeostatic ETSB groups is multifold over ETSB*^Tim−^* groups (Figure 6D). FACS further verified that ETSB*^Tim−^* could not elevate in situ Vγ4*γδ*TCR/central naïve TCR index (Figure 6E), meaning lymphocytes in thymic rudiment without TCR orchestration. As for the preparation of Arntl‐Tim‐low/negative ETSB (ETSB*^Tim−^*), functional blockade to Clock/Arntl‐Tim feedback loop by liposomal quercetin modifying 3D‐ETSB was adopted since it is hard for Arntl/Tim‐null OSEs (OSEs*^Tim−^*) to remodel EMT‐3D‐spheroids (Figure S6A, Supporting Information). About 250 3D‐floating ETSB spheroids per ml were exposed to 15 µg of Lipo‐Que for 12 h to obtain Tim‐low/negative ETSB (Figure S6B, Supporting Information). Too early (day‐0/‐7) Lipo‐Que modification for Clock/Arntl‐Tim regulatory loop would hinder the conversion from OSEs into 3D‐EMT spheroids (Figure S6C, Supporting Information). Yet after 3D conversion, Lipo‐Que modification would not impede the expression of Nanog and Oct‐4 in 3D‐ETSB*^Tim−^*(Figure S6D, Supporting Information). WGCNA indicated that ETSB would resettle Clock/Arntl‐Per3/Tim‐LTβR‐NF‐κB‐RelA/B molecule cascades to re‐prime central‐thymus/peripheral‐defense networks evolved towards endogenous innovation yet relevant modification could impeded the feedback loops (Figure S6E, Supporting Information).

### 2.5 ETSB‐Innovated Attack‐Defense Dynamics Against Peripheral Biologic Challenges

**Figure 7:**
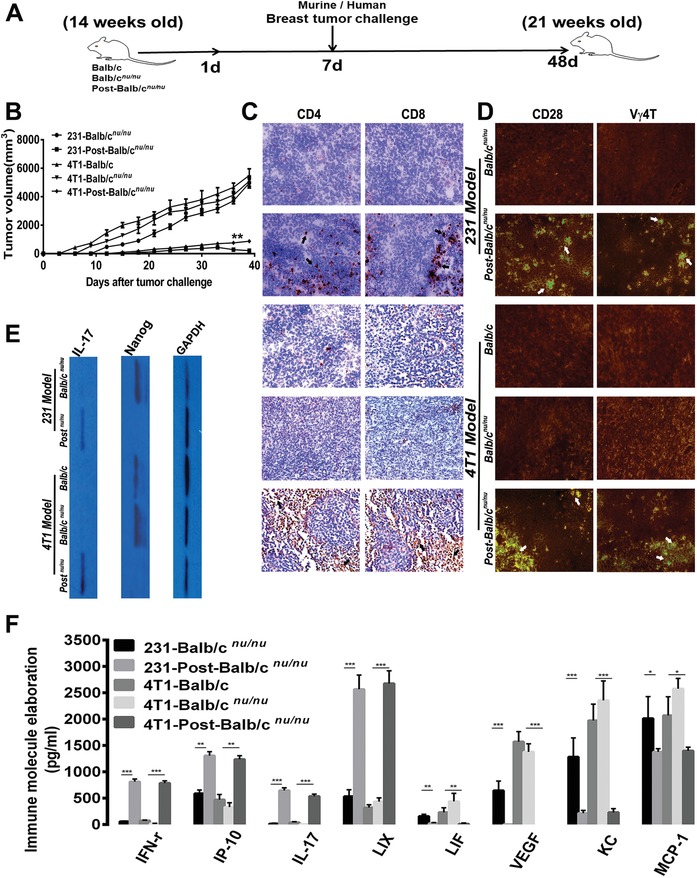
ETSB‐innovated attack‐defense dynamics impedes peripheral tumor‐renewal signaling. A) Study overview of human or murine mammary tumor challenges to hETSB‐inoculated (Post‐Balb/c*^nu/nu^*) with Balb/c*^nu/nu^* and normal Balb/c as control hosts (*n* = 10). B) Graph depicts the tumorigenesis dynamics of five groups from independent human 231 and murine 4T1 models based on tumor volumes at indicated days after tumor challenges. C) Micrographs taken at 200× magnification illustrate peripheral tumor parenchyma of different groups, with renovated T cell microenvironment indicated by black arrows. Data show parts of representative images from five groups in two independent mammary models. D) As peripheral cell microenvironment, CD28 and Vγ4T subsets congregating into local tumor nests were monitored by immunofluorescence dynamic scanning, with positive subsets indicated by white arrows. E) Immunoblotting was used to detect in situ tumor‐renewal signaling Nanog and local immune molecule IL‐17 expression in five groups from two independent models, with GAPDH as internal standards. F) As immunoregulatory microenvironment monitored by the magnetic bead microarray, the regulated molecules cover IFN‐γ, IP‐10 (IFN‐γ‐inducible protein 10, CXCL10), IL‐17, LIX/CXCL5 (LPS‐induced CXC chemokine), LIF (Leukemia inhibitory factor), VEGF, KC (keratinocyte‐derived chemokine) and MCP‐1/CCL2 (monocyte chemoattractant protein1). Data represent the summary of five groups from two independent mammary models. Values expressed as means±SD. *P < 0.05; **P < 0.01; ***P < 0.005. See also Figures S7–S8 (Supporting Information).

Normal Balb/c, Balb/c*^nu/nu^* and Post‐Balb/c*^nu/nu^* were, respectively, subjected to independent human (MDA‐231) or murine (4T1) mammary tumor challenges at week 15 (**Figure** **7**A), and then allowed for a 6 week observation period. Different groups from two independent models have distinct tumorigenesis dynamic profiles (Figure 7B). By 200× magnification (Figure 7C) and dynamic immunofluorescence scanning (Figure 7D) for tumor parenchyma of two independent models, plentiful CD4/8/CD28 and Vγ4T‐cell subsets congregating into local tumor nests were monitored in Post‐Balb/c*^nu/nu^*, yet very few in Normal Balb/c or Balb/c*^nu/nu^* groups as without TCR‐innovated peripheral microenvironment. Immunoblotting has detected in situ tumor‐renewal signaling Nanog in Normal Balb/c and Balb/c*^nu/nu^* groups and local IL‐17 expression in Post‐Balb/c*^nu/nu^* groups (Figure 7E). As peripheral immunoregulatory microenvironment detected by magnetic bead microarray, collective enhancement of regulatory molecules covers IFN‐γ, IP‐10 (IFN‐γ‐inducible protein 10, CXCL10), IL‐17, LIX/CXCL5 (LPS‐induced CXC chemokine), LIF (Leukemia inhibitory factor); selective down‐regulation of molecules includes VEGF, KC (keratinocyte‐derived chemokine) and MCP‐1/CCL2 (monocyte chemoattractant protein1) in Post‐Balb/c*^nu/nu^* groups from two independent models (Figure 7F). Post‐Balb/c*^nu/nu^* hosts were established as described above and received three kind human tumor challenges at week 15 (Figure S7A, Supporting Information), and then allowed for a 6‐week observation period for tumor growth and metastasis. Body weight curves of ETSB‐inoculated groups keep paralleling closely that of Control group, with no significant differences among them (Figure S7B, Supporting Information). Experiment was repeated in three independent models with similar results and breast tumor model was specified as the chief resource of data analysis. Tumorigenesis dynamics has been evidently reversed by TCR‐innovated defense dynamics (Figure S7C, Supporting Information). Dynamic progression of different tumor burdens has been deterred to full recession in ultimate stage in ETSB groups, where 0% of tumor‐free survival rate in midway stage were enhanced to about 80% in ultimate stage (Figure S7D, Supporting Information). Meanwhile, metastatic neoplastic nests (Figure S7E, Supporting Information, upper panel) in lungs are proliferating stably in Control/CB groups, yet have been reversed toward eventual regression in ETSB groups, especially, with no microscopic toxic or cell‐deposited clinical/pathologic indications in pulmonary alveoli. Metastasis nodules in draining sentinel LNs (Figure S7E, Supporting Information, lower panel, Control/CB groups) have subsided to eventual regression in ETSB groups. Histomorphometry verifies that percent metastasis index into lungs and LNs could be evidently reversed into ultimate zero metastasis growth in ETSB groups (Figure S7F, Supporting Information). In addition, no systemic toxicity or cell‐deposited pathologic lesions is detected in heart, liver, or kidney from ETSB inoculation through the experiment till study termination (Figure S7G, Supporting Information). The metastasis dynamics into heart/liver has been evidently reversed toward ultimate zero by ETSB‐innovated attack‐defense dynamics (Figure S7H, Supporting Information). Additional Post‐BALB/c*^nu/nu^* hosts were established as described above and injected three times around 231‐tumor challenges with neutralizing mAb of anti‐*αβ*TCR, anti‐Vγ1TCR/‐Vγ4TCR, or anti‐CD28, with normal IgG as control Ab to see if ETSB‐primed peripheral defense axis revival would be deterred by TCR blockade (Figure S8A, Supporting Information). Tumor‐free induction by ETSB could be evidently terminated by *αβ*TCR, CD28, single Vγ4TCR, or Vγ4/1TCR elimination, yet not by single Vγ1TCR depletion (Figure S8B, Supporting Information). Vγ4*γδ*T cell blockade could boost tumor metastatic dynamics to lungs and LNs in ETSB‐inoculated hosts (Figure S8C, Supporting Information). Magnetic bead microarray was adopted to monitor immunoregulatory microenvironment of Post‐BALB/c*^nu/nu^* after TCR blockade (Figure S8D, Supporting Information). Regulated molecules cover IFN‐γ, IP‐10/CXCL 10, IL‐17, LIX/CXCL5, LIF, VEGF, KC and MCP‐1/CCL2. Especially, IFN‐γ and IL‐17 levels have been substantially downregulated by the Vγ4/1TCR blockade. Consequently, ETSB could reoriginate molecule clock to reset voided thymic rudiment into endogenous renovation by Clock/Arntl‐Per3/Tim loop re‐priming TECs into MET/EMT dynamic reversion in central microenvironment for thymic progenitors enriched and subjected to systemic innovation toward TCR‐evolving orchestration, with innate‐adaptive defense dynamics revival against progressive biologic burden and tumor challenges. Where *αβ*T‐dominated evolving‐subsets could address nonstem terminal cancer‐cell subsets rapidly; Vγ4TCR‐dominated evolving‐subsets can accurately eliminate peripheral EMT/CSC‐evolving pools, which is thereby able to fully address therapy‐resistance and relapse‐metastasis (Figure S8E, Supporting Information).

## 3 Discussion

When thymic function is compromised, there would be an inevitable tendency into immune dysregulation ranging from immunodeficiency and opportunistic infections, through to increased incidence of autoimmune disorders and cancer development. Therefore, it is crucial to drive endogenous thymic renovation so as to extricate from such dilemmas completely. However, without resetting thymus‐voided bioclock, such ideal modalities as evoking defective/aging thymus from involution into evolutionary revival would face formidable puzzles, especially to therapeutic modulation of hosts with advanced cancer. It is a pity that not all adult individuals could share normal functional thymus glands, which has yet to be satisfactorily explained. Early thymus shares the same rudiment with parathyroid for development,^4^ with original functional roles in enhancing reproductive system and adolescence growth, which gives obvious evidence as an erstwhile endocrine organ.^5^, ^6^, ^7^ Namely, T‐cell production may not be the original function of the thymus, but rather a recently evolved function. Late acquisition of secondary lymphopoietic function by thymus may also explain why T cells (rather than B cells) are the only blood lineage first produced ectopically to bone marrow, then migrated to and developed in an epithelial (rather than mesenchymal) organ, a feature of T‐cell biology for which the relevant basis has not been satisfactorily explained. Untimely thymic involution may represent incomplete adaption for an erst still‐evolving endocrine organ to biological burden of lymphopoiesis. Before puberty, thymic parenchyma possesses harmonious central‐microenvironments that play obligate roles in supporting various aspects for T‐cell repertoire orchestration and selection. Thymic cortex offers microenvironments that promote differentiation of lymphoid progenitor cells into T‐lineage cells and positively select a diverse and functional‐TCR repertoire, whereas thymic medulla provides microenvironments that elicit self‐tolerance by deleting self‐reactive T cells. Why adult (after puberty before senile) individual is usually equipped with a defective (rather than dynamic) thymus may not represent a biological natural selection, but rather meaning integral inadaptation to evolutionary dynamics from erstwhile endocrine towards mature immune organ. Our last work has found that thymus involution in adult primates could be reversed to harmonizing revival of dynamic rhythm by 3D‐biologics, implicating incomplete adaptation of adult thymic rhythm dynamics to lymphopoietic activity as biological burden, rather than natural biological selection.

Young thymus with dynamic rhythm provides superior cell and humoral microenvironment for renewable T‐cell subset development and mature.^20^, ^21^ TECs, similar to OSEs, would transition back and forth between epithelial–mesenchymal phenotypes (intrathymic EMT/MET) in tissue architecture conversion covering renewal/evolution development/organogenesis, or senescence/involution. Similarly, EMT/MET conversion‐niche could also serve as in vivo hotbed of development/growth and selection/evolution for tumor cells via adjustment and control at cellular transcriptional level. Consequently EMT/stem cell transition‐based renewable 3D biologics could adjust crucial cell transcription and molecule rhythm to reset central microenvironment for T‐cell repertoire revival targeted at constantly evolving EMT/CSC‐pool to fully resolve current dilemma.^22^ In our study, OSE‐derived 3D‐ETSB primes invalid thymic rudiment (original parathyroid‐like vesicles) of immune deficient/aging hosts into evolutionary in situ renewal mainly via bHLH‐Clock/Arntl‐Per/Tim signal transcription served as dynamic bioclock resetting central microenvironments, which further pioneers 3D‐panoramic cortex/medulla revival by TECs undergoing MET/EMT reversion under feedback modulation of LTβR‐NF‐κB‐RelA/B‐Wnt signaling cascades, with dynamic evolution of defective immune core and final development of defense dynamics. Most notably, unbiased and nonpresumptive transcriptome analyses, using informatics approaches, strongly implicate Clock/Arntl‐Per3/Tim with LTβR‐NF‐κB‐RelA/B‐TCR signaling‐networks involved in TEC‐innovated MET/EMT/lymphopoietic microenvironments revival, with integral changes occurring in most components of the loops virtually. The resettled core microenvironments into panoramic revivals cover Clock‐Arntl‐Per‐Tim/LTβR‐NF‐κB signals resetting molecule microenvironments undergoing dynamic transcription and TECs undergoing MET/EMT reoriginating such cell microenvironments as renewal‐hotspots, with evolutionary dynamic rhythm for endogenous progenitor cells innovating Vγ4*γδ*T‐dominated T‐lineage cells and γIFN/IL‐17 expression reactivity against biologic burden. Thus, Clock‐Tim/NF‐κB loops have played important roles in shaping the immune system for deficient hosts with more relevant gene expression involved crucially in re‐priming voided‐microenvironments into evolutionary transcription, and is essential for lymphocyte mature to provoke innate‐adaptive responses against diverse invading pathogens and the constantly evolving EMT/CSC‐pool in tumor hosts. As *γδ*T cells are able to quickly produce IL‐17 and IFN‐γ in response to a variety of stimuli, they have linked innate and adaptive immune responses.^23^, ^24^ Relatively little is known about their development, but recently their conversion into IL‐17/γIFN producing cells has been reported to be imprinted early in developing *γδ* thymocyte progenitors to Vγ4*γδ*T‐dominated T cell repertoire, correlating with expression of TNF receptor members in integral LTβR‐NF‐κB‐TCR network renewal.^25^ Presence of Clock‐Arntl/Per3‐Tim/CD27/LTβRs‐CD28 feedback loop suggests vital role in classical/alternative pathways innovating core microenvironment for evolutionary MET/EMT‐undergoing TECs with dynamic rhythm to prime *γδ*TCR toward *αβ*TCR with enhanced γIFN/IL‐17 reactivity. This study unveils distinct T‐cell intrinsic roles of RelA and RelB in NF‐κB family members, for RelA in accessory thymocytes and RelB in thymic *γδ*‐T cell progenitors, as specific requirements to provoke peripheral T cells to rapidly produce IL‐17/γIFN, reoriginating peripheral‐immune axis to fight EMT/CSC subsets or other biological burdens such as bacterial infections. Despite remarkable progress in understanding role of Clock/Per3‐Tim/NF‐κB cascades in normal lymphocyte development and function, much still remains to be learned. As core circadian rhythm gene in *Drosophila*, Tim is retained in mammals but has no apparent mammalian circadian clock function. Such elucidated areas in need of greater attention include gaining a better understanding of why Clock‐Arntl/Per3‐Tim feedback axis by LTβR‐NF‐κB‐TCR pathway modulates orchestration of innate T‐cell lineages, determining what roles the Clock pathway serves in thymocyte precursors maturation, and cataloging the many and varied roles the Clock/Arntl‐Per3/Tim loop serves in thymic precursors during aging‐thymus revival.^26^ So far, most reports about in vivo thymic regenerations in athymic nude mice were based on thymic cells or tissue‐grafts transplantation rather than on endogenous revival from voided‐rudiment in situ. However, hosts post undergoing exogenous thymus transplantation may easily develop autoimmune diseases or other immune dysregulations due to incompatibility of alien thymic tissues to recipient innate progenitors.^27^ Here, it is an innovative report to unravel thymic 3D‐evolution instance from endogenous voided rudiment into panoramic cortex/medulla revival in immune‐deficient/aging hosts via dynamic modulation involved crucially in Clock‐Arntl/Per3‐Tim cascade yet little in foxn1‐related factors. Since central immune retrogression with peripheral T‐subsets depletion is the key mechanism shared by the victims bearing progressive cancer and many immune‐disharmonies covering AIDS and other senility‐related disorders,^28^, ^29^, ^30^ our study may pioneer a generalizable therapeutic‐modality for such disorders via evoking panoramic innovation of thymus‐related core‐rhythm dynamics and peripheral immune‐dynamics.

## 4 Experimental Section

Methodology is described in Supporting Information.^31^, ^32^, ^33^, ^34^, ^35^, ^36^, ^37^, ^38^

## Conflict of Interest

The authors declare no conflict of interest.

## Supporting information

Supplementary

Supplementary

Supplementary

Supplementary

Supplementary

## References

[1] N. Bredenkamp , S. Ulyanchenko , K. E. O'Neill , N. R. Manley , H. J. Vaidya , C. C. Blackburn , Nat. Cell Biol. 2014, 16, 902. 25150981 10.1038/ncb3023 PMC4153409

[2] L. DeFrancesco , Nat. Biotechnol. 2012, 30, 411. 10.1038/nbt.2446 23222776

[3] D.‐M. Su , D. Aw , D. B. Palmer , Curr. Opin. Immunol. 2013, 25, 498. 23809359 10.1016/j.coi.2013.05.018

[4] A. Graham , Am. J. Med. Genet., Part A 2003, 119A, 251. 12784288 10.1002/ajmg.a.10980

[5] H. Besedovsky , E. Sorkin , Nature 1974, 249, 356. 4858269 10.1038/249356a0

[6] Y. Weinstein , Mech. Ageing Dev. 1978, 8, 63. 357852 10.1016/0047-6374(78)90007-6

[7] W. Pierpaoli , E. Sorkin , Nature 1972, 238, 282. 4507169 10.1038/newbio238282a0

[8] H. E. Lynch , G. L. Goldberg , A. Chidgey , M. R. M. V. D. Brink , R. Boyd , G. D. Sempowski , Trends Immunol. 2009, 30, 366. 19540807 10.1016/j.it.2009.04.003 PMC2750859

[9] S. Siddiqui , A. Lustig , A. Carter , M. Sankar , C. M. Daimon , R. T. Premont , H. Etienne , J. van Gastel , A. Azmi , J. Janssens , Aging 2017, 9, 706. 28260693 10.18632/aging.101185 PMC5391227

[10] A. M. Holland , M. R. van den Brink , Curr. Opin. Immunol. 2009, 21, 454. 19608394 10.1016/j.coi.2009.06.002 PMC2731988

[11] R. Zachariah , N. Ford , M. Philips , S. Lynch , M. Massaquoi , V. Janssens , A. Harries , Trans. R. Soc. Trop. Med. Hyg. 2009, 103, 549. 18992905 10.1016/j.trstmh.2008.09.019

[12] A. Lepletier , A. P. Chidgey , W. Savino , Gerontology 2015, 61, 504. 25765703 10.1159/000375160

[13] Y. Hamazaki , M. Sekai , N. Minato , Immunol. Rev. 2016, 271, 38. 27088906 10.1111/imr.12412

[14] N. M. Alajez , J. Schmielau , M. D. Alter , M. Cascio , O. J. Finn , Blood 2005, 105, 4583. 15746083 10.1182/blood-2004-10-3848 PMC1894994

[15] A. Nuzhat , E. W. Thompson , M. A. Quinn , J. Cell. Physiol. 2010, 213, 581. 10.1002/jcp.21240 17708542

[16] A. Banerjee , A. Vasanthakumar , G. Grigoriadis , Immunol. Cell Biol. 2013, 91, 340. 23567897 10.1038/icb.2013.12

[17] W. V. Ewijk , B. P. Wang , G. Hollander , H. Kawamoto , E. Spanopoulou , M. Itoi , T. Amagai , Y. F. Jiang , W. T. V. Germeraad , W. F. Chen , Semin. Immunol. 1999, 11, 57. 9950752 10.1006/smim.1998.0158

[18] V. Tirino , V. Desiderio , F. Paino , A. De Rosa , F. Papaccio , M. La Noce , L. Laino , F. De Francesco , G. Papaccio , FASEB J. 2013, 27, 13. 23024375 10.1096/fj.12-218222

[19] J. Kim , W. A. Li , Y. Choi , S. A. Lewin , C. S. Verbeke , G. Dranoff , D. J. Mooney , Nat. Biotechnol. 2015, 33, 64. 25485616 10.1038/nbt.3071 PMC4318563

[20] M. Li , K. Guo , S. Ikehara , Int. J. Mol. Sci. 2014, 15, 19226. 25342318 10.3390/ijms151019226 PMC4227270

[21] S. L. Shiao , A. P. Ganesan , H. S. Rugo , L. M. Coussens , Genes Dev. 2011, 25, 2559. 22190457 10.1101/gad.169029.111 PMC3248678

[22] T. T. Smith , J. C. Roth , G. K. Friedman , G. Y. Gillespie , Oncolytic Virother. 2014, 2014, 21. 24834430 10.2147/OV.S52749 PMC4018757

[23] K. D. Jensen , Y.‐h. Chien , Curr. Opin. Immunol. 2009, 21, 140. 19321327 10.1016/j.coi.2009.02.008 PMC2697822

[24] S. Kensuke , Y. Hisakata , N. Masataka , H. Shinya , K. Yoshinori , K. Ryo , Y. Yasunobu , J. Immunol. 2014, 192, 2210.24489104

[25] J. C. Ribot , D. J. Pang , J. F. Neves , V. Peperzak , S. J. Roberts , M. Girardi , J. Borst , A. C. Hayday , D. J. Pennington , B. Silva‐Santos , Nat. Immunol. 2009, 10, 427. 19270712 10.1038/ni.1717 PMC4167721

[26] X. Yang , P. A. Wood , W. J. M. Hrushesky , J. Biol. Chem. 2010, 285, 3030. 19996108 10.1074/jbc.M109.050237 PMC2823431

[27] S. Sakaguchi , N. Sakaguchi , J. Exp. Med. 1988, 167, 1479. 2965739 10.1084/jem.167.4.1479 PMC2188920

[28] R. Reyes , D. R. Canfield , U. Esser , L. A. Adamson , C. R. Brown , C. Cheng‐Mayer , M. B. Gardner , J. M. Harouse , P. A. Luciw , J. Virol. 2004, 78, 2121. 14747577 10.1128/JVI.78.4.2121-2130.2004 PMC369416

[29] Y. Zhang , H. Yang , Q. Li , X. Duan , X. Zhao , Y. Wei , X. Chen , J. Immunol. 2018, 201, 1975. 30150284 10.4049/jimmunol.1701727

[30] K. Alves , M. Canzian , E. Delwart , AIDS Res. Hum. Retroviruses 2002, 18, 161. 11839149 10.1089/08892220252779700

[31] S. Barlow , G. Brooke , K. Chatterjee , G. Price , R. Pelekanos , T. Rossetti , M. Doody , D. Venter , S. Pain , K. Gilshenan , Stem Cells Dev. 2008, 17, 1095. 19006451 10.1089/scd.2007.0154

[32] M. Evangelista , M. Soncini , O. Parolini , Cytotechnology 2008, 58, 33. 19002775 10.1007/s10616-008-9162-z PMC2593758

[33] R. Hisazumi , M. Kayumi , W. Zhang , R. Kikukawa , T. Nasu , M. Yasuda , Vet. Immunol. Immunopathol. 2016, 169, 74. 26827842 10.1016/j.vetimm.2015.12.009

[34] R. Hisazumi , M. Kayumi , R. Kikukawa , T. Nasu , M. Yasuda , Vet. Immunol. Immunopathol. 2015, 167, 86. 26143006 10.1016/j.vetimm.2015.06.010

[35] Z. P. Yuan , L. J. Chen , L. Y. Fan , M. H. Tang , G. L. Yang , H. S. Yang , X. B. Du , G. Q. Wang , W. X. Yao , Q. M. Zhao , B. Ye , R. Wang , P. Diao , W. Zhang , H. B. Wu , X. Zhao , Y. Q. Wei , Clin. Cancer Res. 2006, 12, 3193. 16707620 10.1158/1078-0432.CCR-05-2365

[36] Q. Long , Y. Xie , Y. Huang , Q. Wu , H. Zhang , S. Xiong , Y. Liu , L. Chen , Y. Wei , X. Zhao , C. Gong , J. Biomed. Nanotechnol. 2013, 9, 965. 23858960 10.1166/jbn.2013.1596

[37] B. H. Kim , S. M. Cho , A. M. Reddy , Y. S. Kim , K. R. Min , Y. Kim , Biochem. Pharmacol. 2005, 69, 1577. 15896337 10.1016/j.bcp.2005.03.014

[38] A. J. Moreira , C. Fraga , M. Alonso , P. S. Collado , C. Zetller , C. Marroni , N. Marroni , J. Gonzalez‐Gallego , Biochem. Pharmacol. 2004, 68, 1939. 15476665 10.1016/j.bcp.2004.07.016

