# Supplementary material for: Transcription‐Related Dynamics from Immune Disability into Endogenous Innovation
Source: Adv Sci (Weinh). 2019 Sep 30;6(23):1900767. doi: 10.1002/advs.201900767 (PMC6891922; doi:10.1002/advs.201900767)
Supplement: Supplementary file 1 — Supplementary [file ADVS-6-1900767-s002.pdf]

Copyright WILEY-VCH Verlag GmbH & Co. KGaA, 69469 Weinheim, Germany, 2019.

Supporting Information

for *Adv. Sci.,* DOI: 10.1002/advs.201900767

Transcription-Related Dynamics from Immune Disability into Endogenous Innovation

*Yanna Zhang, Qian Li, Panyan Hou, Yanan Lu, Huanhuan Yang, Xiaojuan Lin, Chao Su, Yuquan Wei, Xiulin Yang, Hanshuo Yang, Xia Zhao, and Xiancheng Chen**

Supplemental Figures

Fig.S1

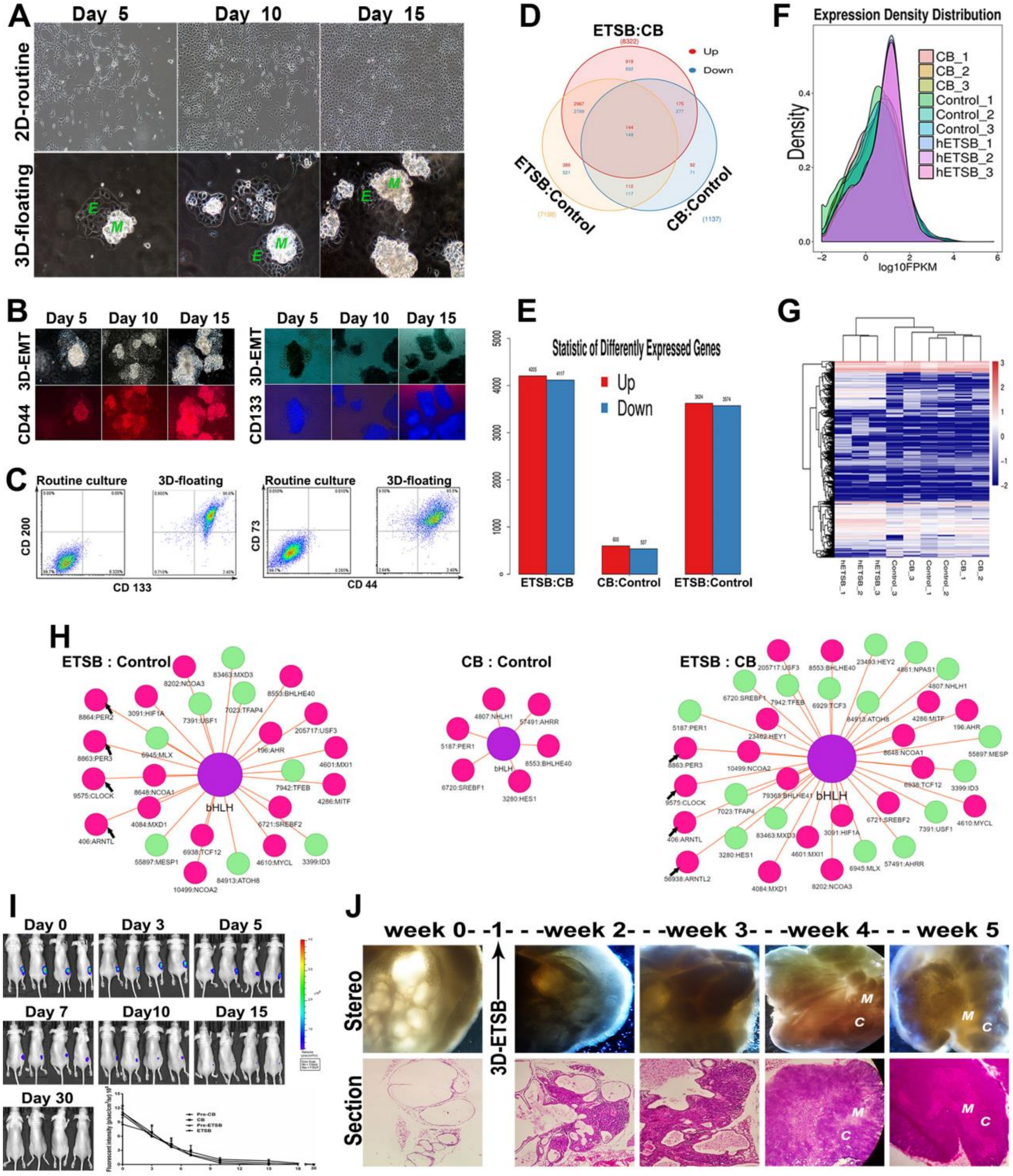

Fig.S1. Transcriptional dynamics and biodistribution of EMT-3D-spheroid. Related to Fig.1.

A, Dynamic comparison between 2D routine culture pattern and 3D-floating culture model.

B, Immunofluorescence dynamic scanning was used to monitor 3D-EMT transition: positive phenotypes for CD44 and CD133 in mesenchymal transition parts but negative in epithelial non-transition parts.

C, FACS assay was adopted to detect the multiepitope (CD44/CD73 and CD133/CD200) expression index for EMT/MET dynamic transition at day 15.

D, Venn analysis based on DEG identifies that both ETSB:CB and ETSB:Control comparisons are involved in much more up-/down-regulated gene transcriptions than CB:Control comparison.

E, Graph depicts the statistical analyses for DEG comparison among groups, with ETSB:CB comparison involving in more up-/down-regulated genes than other comparison.

F, On the distribution graph, each sample with 3 waveforms represents 3 biologic replicates and hETSB waveforms cluster higher expression density than CB and Control.

G, TF expressions were clustered into heat maps. Red represents elevated expression while blue represents decreased expression. ETSB replicates cover both higher expression and closer relatedness.

H, Relational network analyses were adopted for TF-coding ability comparison of bHLH family between two groups. Upregulated transcription of Clock, Per3, Arntl was identified with arrows.

I, ETSB was subcutaneously injected into Balb/c$^{nu/nu}$ nude mice and imaged every other day, showing initial and eventual localization in subcutaneous tissues by day 10 and gradual diminishment until complete disappearance by day 30, with similar result by direct injection of the biologics to thymic rudiments *in situ*.

J, Thymic rudiments from routine nude mice were nursed under ameliorative DMEM/F12/1640-integrated medium with 3D-floating ETSB yet no contact by filter screen and monitored via stereomicroscopy for over four weeks, with final cortex-medulla survived panoramically without direct toxicity.

Fig.S2

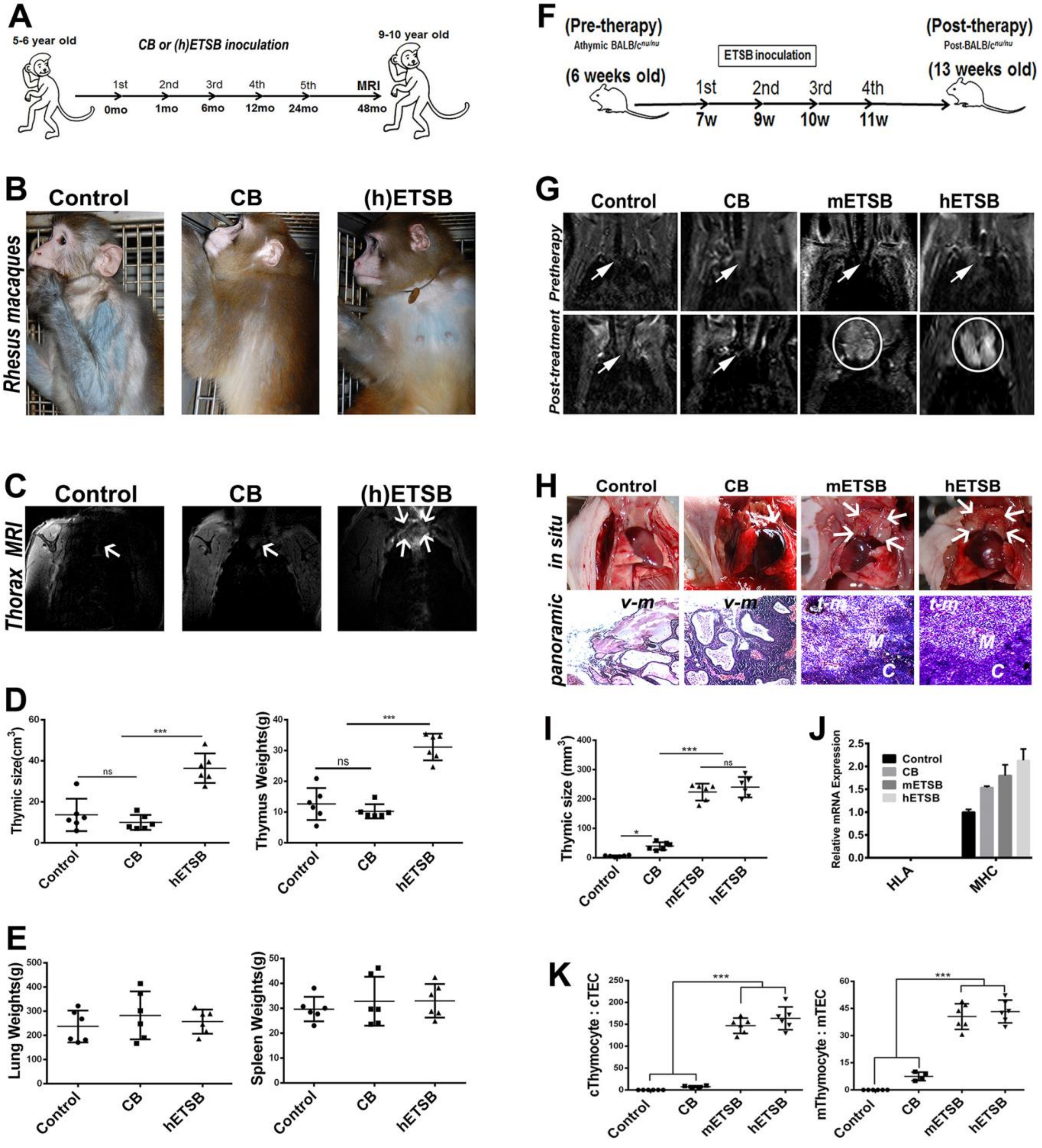
A
5-6 year old
CB or (h)ETSB inoculation
9-10 year old
1st 2nd 3rd 4th 5th MRI
0mo 1mo 6mo 12mo 24mo 48mo
F
(Pre-therapy)
Athymic BALB/c nu/nu
(6 weeks old)
ETSB inoculation
1st 2nd 3rd 4th
7w 9w 10w 11w
(Post-therapy)
Post-BALB/c nu/nu
(13 weeks old)
B
Control CB (h)ETSB
Rhesus macaques
G
Control CB mETSB hETSB
Pretherapy
Post-treatment
C
Control CB (h)ETSB
Thorax MRI
H
Control CB mETSB hETSB
in situ
panoramic
v-m v-m t-m t-m
M C M C
D
Thymic size(cm³)
Thymus Weights(g)
Control CB hETSB
ns ***
I
Thymic size (mm³)
Control CB mETSB hETSB
* *** ns
J
Relative mRNA Expression
Control CB mETSB hETSB
HLA MHC
E
Lung Weights(g)
Spleen Weights(g)
Control CB hETSB
K
cThymocyte : cTEC
mThymocyte : mTEC
Control CB mETSB hETSB
***

Fig.S2. Endogenous innovation for invalid residues with deserted microenvironments. Related to Fig.2.

A, Study strategy for the experimental layout in rhesus. The hosts (5-6-year old rhesus equivalent to 25-36-year old man) were subjected to five times of (h)ETSB inoculations or other corresponding regimens at indicated months during two years (***Month 0-1-6-12-24*** protocol), with MRI or other detections two year later.

B, Photographs show representative appearances among different groups two year after the last experimental inoculation termination.

C, As in (B), MRI was adopted to scan rhesus thorax for different groups two year after experimental procedure termination, with single arrow indicating the sight of atrophied/aging or vanished thymus in front of trachea over heart and multiple-arrows revealing *in situ* evolved thymus(n=6)**.**

D, Graphs represent the thymic size based on tridimensional scan and weight among different groups.

E, Lung and spleen weights keep similar among different groups ($P>0.05$).

F, Schematic depiction for experimental layout in Balb/$c^{nu/nu}$ nude mice.

G, MRI was used as non-invasive detection to scan pre- and post- therapy thorax, with arrows indicating the athymic sign in front of trachea over heart and white circles revealing *in situ* evolved thymus(n=6).

H, *In situ* inspection manifests thoracic cavity in nude mice, with tridimensional-innovation of left/right thymic lobes indicated by four arrows and superficial hyperemia above heart. Panoramic histomorphometry was used in different groups to monitor how parathyroid-like residual in voided microenvironment (*v-m*) was endogenized into cortex (*C*) and medulla (*M*) innovation with tridimensional core microenvironment (*t-m*) revival.

I, Graph depicts thymic size to see whether the voided rudiment would be 3D-renovated among different groups. *****$P<0.05$, ******$P<0.01$, *******$P<0.005$; **J**, qRT-PCR of TECs for MHC/HLA revealed the enhanced MHC yet no HLA expression in (h)ETSB group, thus excluding exogenous TEC conversion(n=6). **K,** Graphs represent the thymocytes/TECs ratio for panoramic medulla (m-) and cortex (c-) of four groups (*******$P<0.005$).

Fig.S3

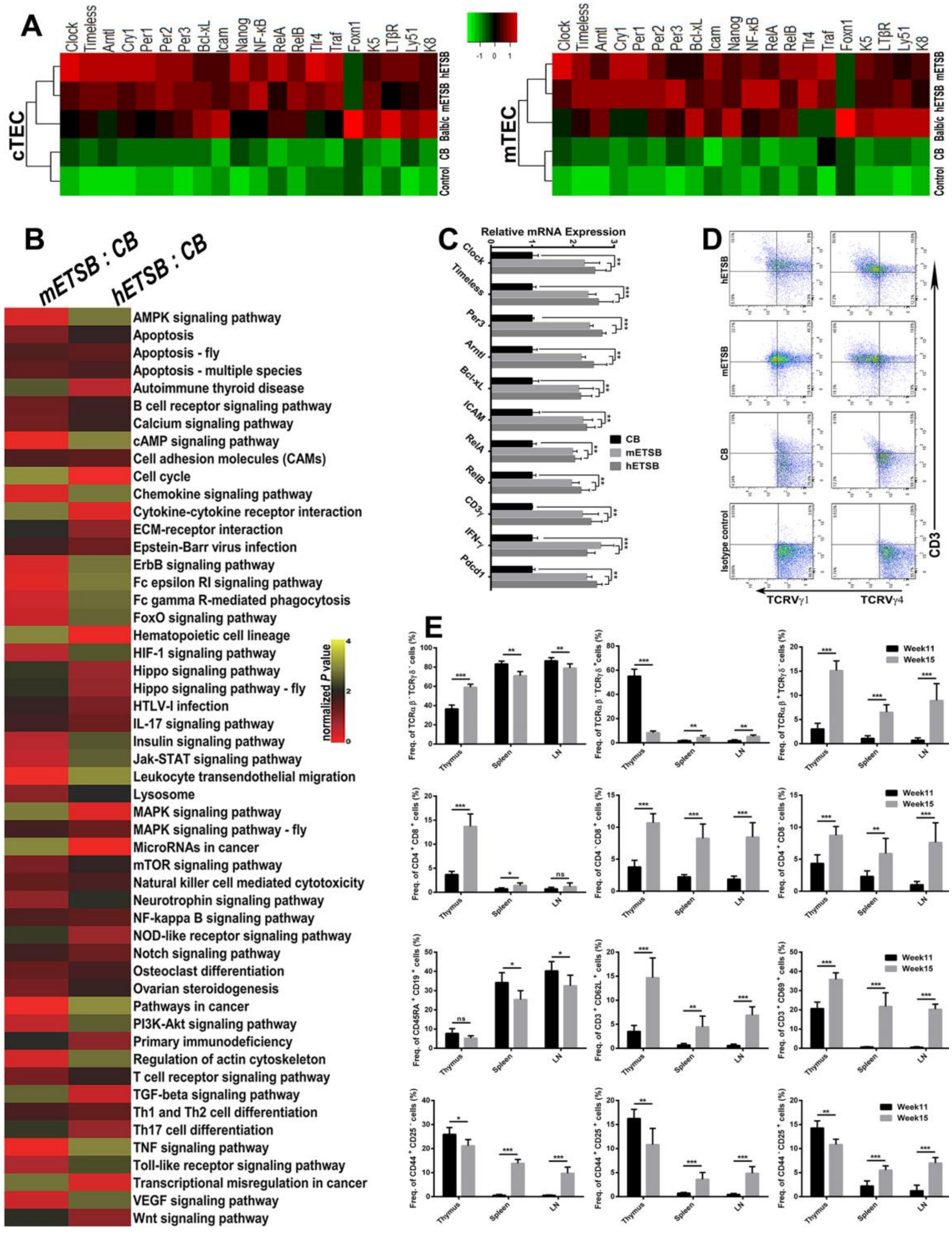
A
cTEC
mTEC
Clock
Timeless
Arntl
Cry1
Per1
Per2
Per3
Bcl-xL
Icam
Nanog
NF-κB
RelA
RelB
Tlr4
Traf
Foxn1
K5
LTβR
Ly51
K8
hETSB
mETSB
Balb/c
CB
Control
B
mETSB : CB
hETSB : CB
AMPK signaling pathway
Apoptosis
Apoptosis - fly
Apoptosis - multiple species
Autoimmune thyroid disease
B cell receptor signaling pathway
Calcium signaling pathway
cAMP signaling pathway
Cell adhesion molecules (CAMs)
Cell cycle
Chemokine signaling pathway
Cytokine-cytokine receptor interaction
ECM-receptor interaction
Epstein-Barr virus infection
ErbB signaling pathway
Fc epsilon RI signaling pathway
Fc gamma R-mediated phagocytosis
FoxO signaling pathway
Hematopoietic cell lineage
HIF-1 signaling pathway
Hippo signaling pathway
Hippo signaling pathway - fly
HTLV-I infection
IL-17 signaling pathway
Insulin signaling pathway
Jak-STAT signaling pathway
Leukocyte transendothelial migration
Lysosome
MAPK signaling pathway
MAPK signaling pathway - fly
MicroRNAs in cancer
mTOR signaling pathway
Natural killer cell mediated cytotoxicity
Neurotrophin signaling pathway
NF-kappa B signaling pathway
NOD-like receptor signaling pathway
Notch signaling pathway
Osteoclast differentiation
Ovarian steroidogenesis
Pathways in cancer
PI3K-Akt signaling pathway
Primary immunodeficiency
Regulation of actin cytoskeleton
T cell receptor signaling pathway
TGF-beta signaling pathway
Th1 and Th2 cell differentiation
Th17 cell differentiation
TNF signaling pathway
Toll-like receptor signaling pathway
Transcriptional misregulation in cancer
VEGF signaling pathway
Wnt signaling pathway
normalized P value
C
Relative mRNA Expression
CB
mETSB
hETSB
Clock
Timeless
Per3
Arntl
Bcl-xL
ICAM
RelA
RelB
CD3γ
IFN-γ
Pdcd1
D
hETSB
mETSB
CB
Isotype control
TCRVγ1
TCRVγ4
CD3
E
Week11
Week15
Thymus
Spleen
LN

Fig.S3. Transcriptional innovation of central molecule and cell microenvironments. Related to Fig.3.

A, Similar and different expressions in TECs of cortex/medulla from different groups covering normal Balb/c were revealed by the heat-map involved crucially in transcriptional factors and active genes associated with Clock modulation and EMT conversion as central immune microenvironment innovation.

B, KEGG for thymic transcriptome from m/hETSB groups versus CB group indicates which signals collectively modulates central molecule microenvironments.

C, Transcriptional indexes of Clock, timeless, Per3, Arntl, Bcl-xL, Icam, RelA, RelB, CD3γ, IFNγ and Pdcd1 of thymocytes in thymic core microenvironment for different groups were identified by qRT-PCR (******P*<0.01; *******P*<0.005; no thymopoiesis for Control).

D, The central-phased *in situ* TCR/CD3 orchestrations for week 12~13 were revealed by FACS assay.

E, Dynamic orchestrations of αβ/γδTCR repertoire for various immune cells in core thymic and peripheral splenic/LN microenvironments in hETSB group during the periods from week 11 to week 15 were further analyzed by FACS assay.

Fig.S4

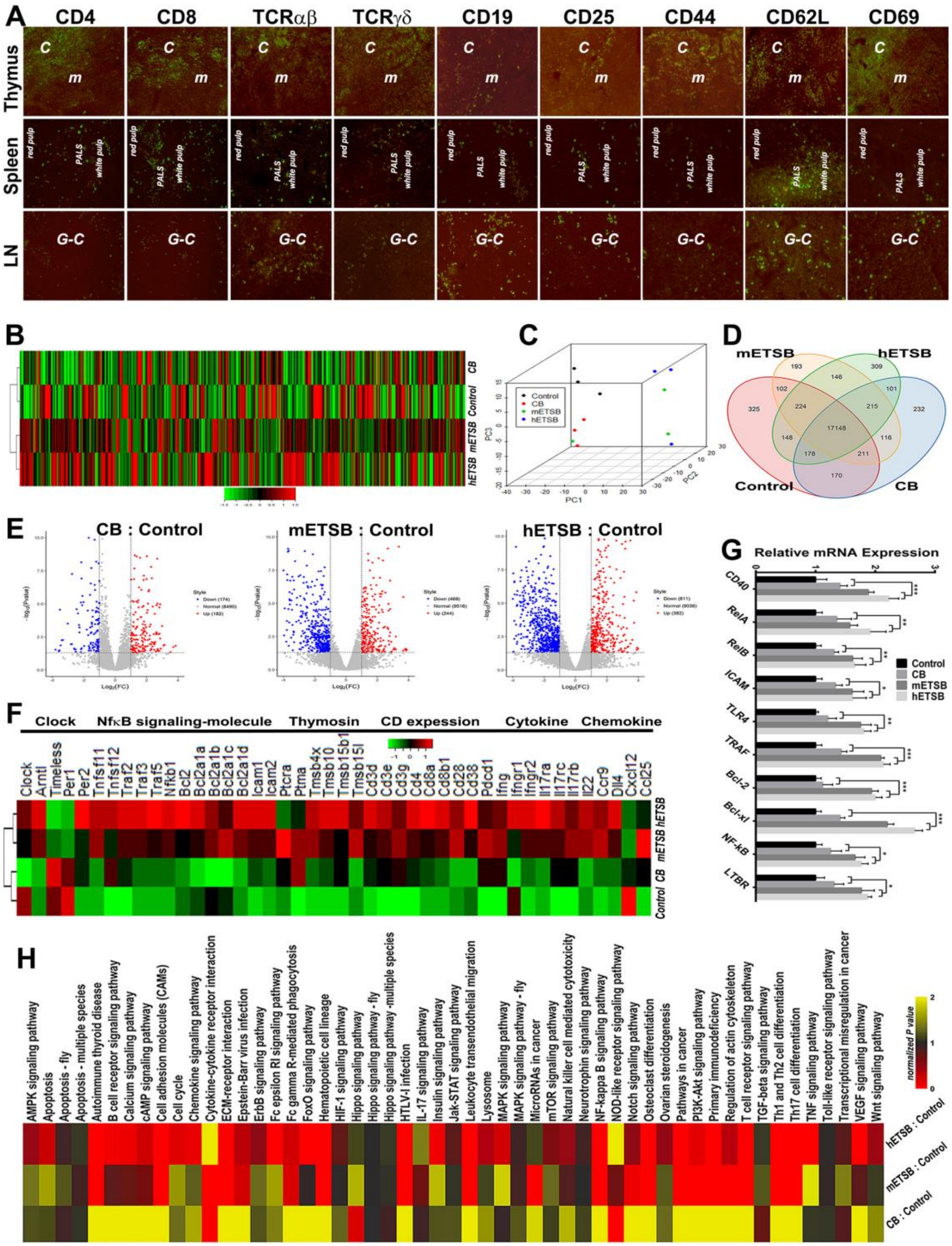
A
CD4
CD8
TCRαβ
TCRγδ
CD19
CD25
CD44
CD62L
CD69
Thymus
Spleen
LN
c
m
red pulp
PALS
white pulp
G-C
B
CB
Control
mETSB
hETSB
C
Control
CB
mETSB
hETSB
PC1
PC2
PC3
D
mETSB
hETSB
Control
CB
E
CB : Control
mETSB : Control
hETSB : Control
G
Relative mRNA Expression
CD40
RelA
RelB
ICAM
TLR4
TRAF
Bcl-2
Bcl-xl
NF-kB
LTBR
Control
CB
mETSB
hETSB
F
Clock
NfκB signaling-molecule
Thymosin
CD expression
Cytokine
Chemokine
H
hETSB : Control
mETSB : Control
CB : Control
normalized P value

Fig.S4. Transcriptional innovation of peripheral cell and molecule microenvironments. Related to Fig.4.

A, Micrographs at 200x confocal magnification illustrate composition and numbers of various immune cells in regenerated thymus (***C***, *in situ* re-originated cortex; ***M***, medulla), spleen (***PALS***, periarterial lymphatic sheath) and LN (***G-C***, germinal centers) in hETSB-inoculated athymic mice of week 14~15.

B, Global expression profiles of different groups are shown as hierarchical clustering heat-maps. Different groups have distinct expression profiles; yet there are close relatives between expression profiles from m- and h- ETSB groups, implicating systemic revival of central-peripheral molecule microenvironments.

C. Splenic transcriptomes were subjected to MDS on expressed genes. Symbols from unorchestrated CB and Control cluster much closely together than symbols from multifunctional ETSB able to address peripheral multiple burdens.

D, Venn diagram based on whole transcriptome for spleen illustrates overlapping distribution of gene expression among Control, CB, mETSB and hETSB groups.

E, Volcano plots represent splenic DEGs between two group comparisons (CB:Control; mETSB:Control; hETSB:Control), with maximum adjustment in hETSB:Control.

F, FPKM-normalized identification of DEGs in splenocytes was displayed as heat-maps to see which feedback regulatory loops or signal cascades were involved crucially in peripheral immune microenvironment modulation.

G, qRT-PCR has revealed the transcriptional index of CD40, RelA, RelB, Icam, Tlr4, Traf, Bcl-2, Bcl-xL, NF-κB and LTβR in splenic peripheral microenvironment of different groups (*******P*<0.01; ********P*<0.005).

H, KEGG for splenocytes from m/hETSB or CB groups versus Control group identifies that networks involved commonly in peripheral molecule microenvironment revival cover NF-κB, Wnt, TCR, Th1/Th2/Th17 differentiations, NK mediated cytotoxicity, Leukocyte transendothelial migration and Cell adhesion molecules (CAMs) signals. See also Video 4.

Fig.S5

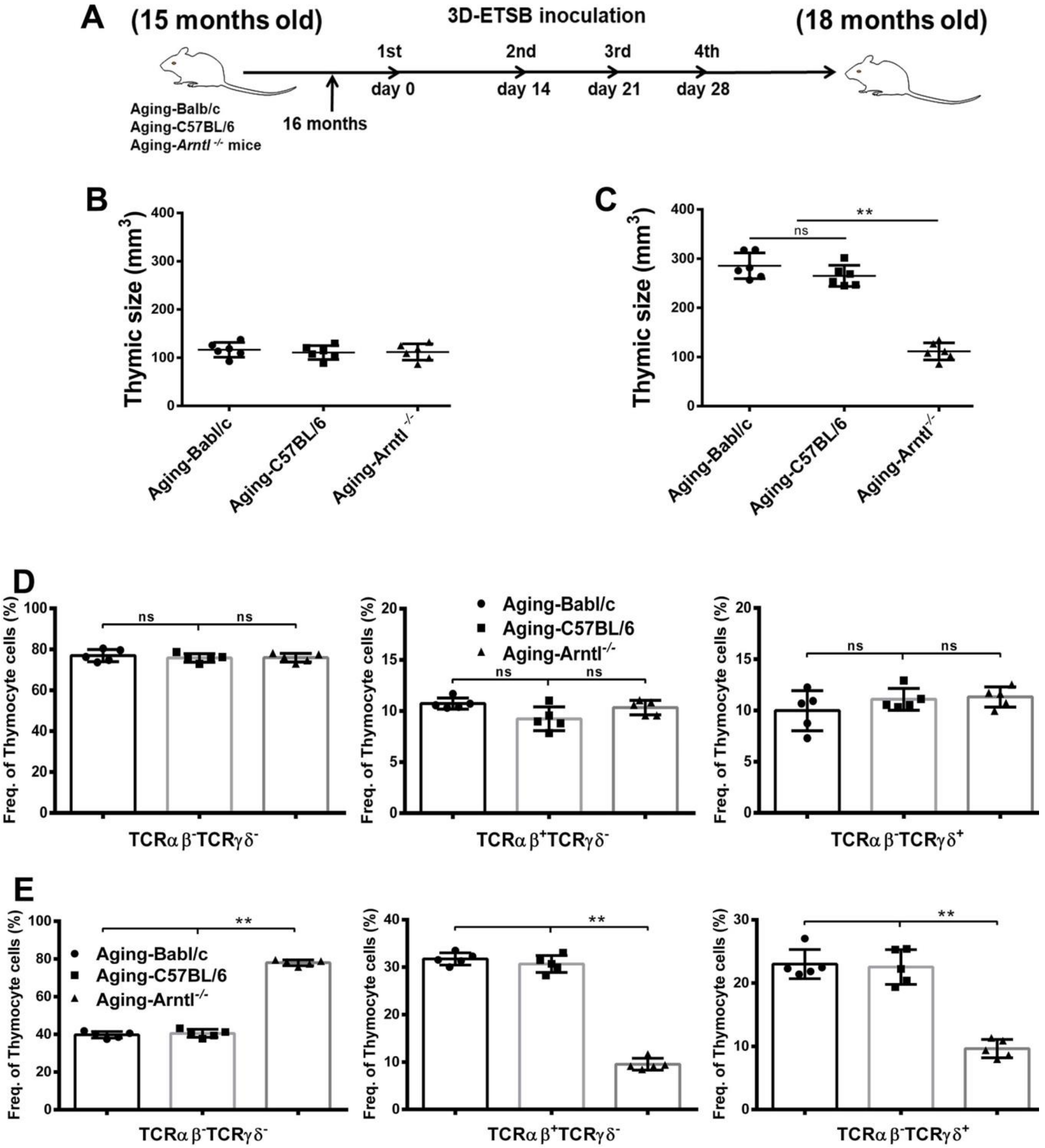
A
(15 months old)
3D-ETSB inoculation
(18 months old)
1st
2nd
3rd
4th
day 0
day 14
day 21
day 28
16 months
Aging-Balb/c
Aging-C57BL/6
Aging-Arntl -/- mice
B
Thymic size (mm3)
Aging-Babl/c
Aging-C57BL/6
Aging-Arntl -/-
C
ns
**
D
Freq. of Thymocyte cells (%)
Aging-Babl/c
Aging-C57BL/6
Aging-Arntl-/-
TCRαβ-TCRγδ-
TCRαβ+TCRγδ-
TCRαβ-TCRγδ+
E

Fig. S5. Aging-thymus revival requires Clock/Arntl-Tim dynamic modulation. Related to Fig.5.

A, Relevant experimental layout in aging *Arntl*$^{-/-}$ hosts with aging Balb/c and C57BL/6 mice as control.

B, Graphs represent the thymic size of aging hosts before ETSB administration at month-16.

C, Graphs represent the thymic size based on aging hosts after ETSB administration at month-18. n=6.

D, FACS assay for thymocytes illustrates the central-phased *in situ*/naïve-αβ/γδTCR index of aging hosts before ETSB administration.

E, FACS assay for thymocytes further illustrates the central-phased *in situ*/naïve-αβ/γδTCR index week 1~2 after 4th ETSB administration so as to compare TCR defense dynamics of aging hosts among different groups. n=5.

Fig.S6

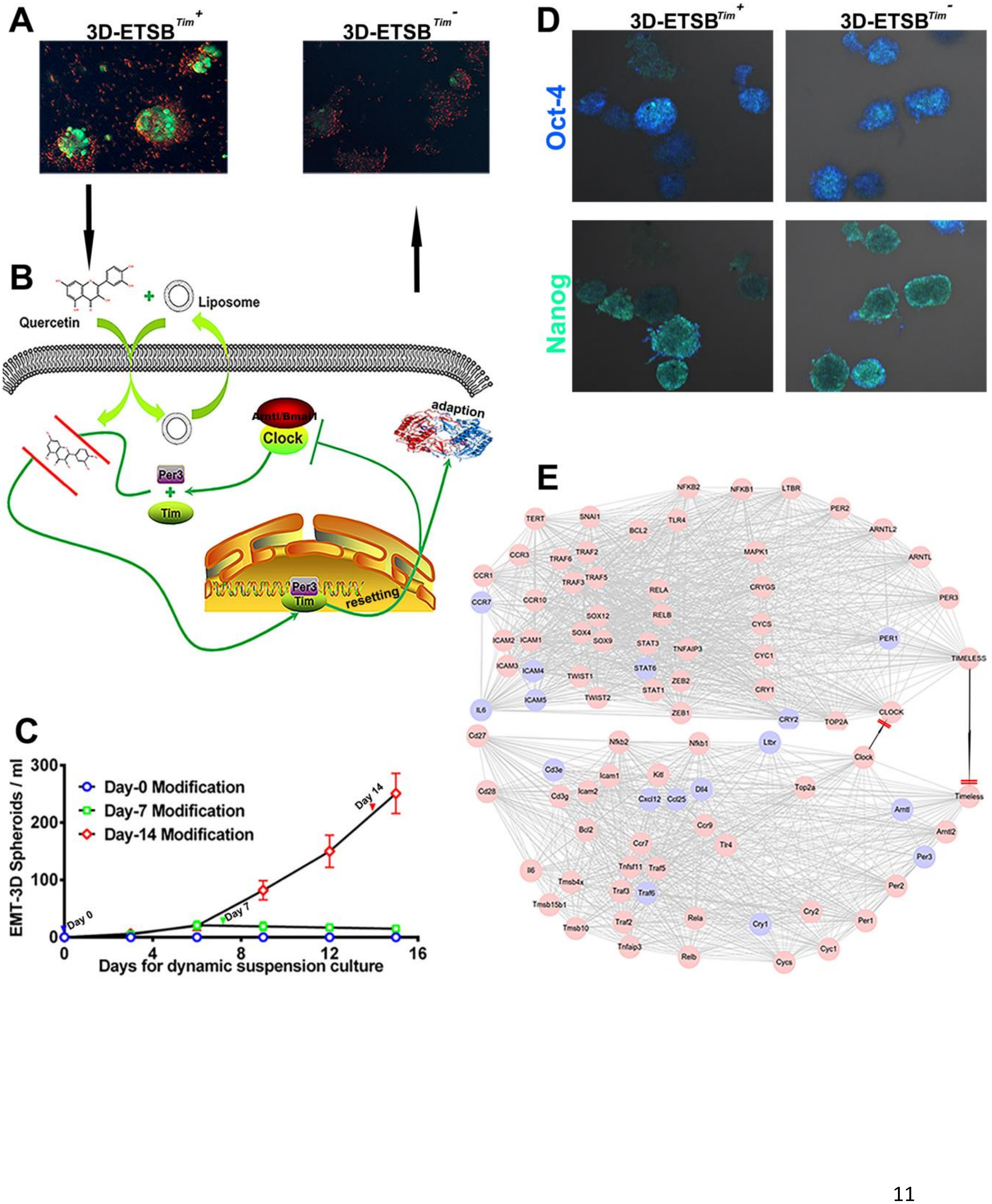
A
3D-ETSB^Tim+
3D-ETSB^Tim-
B
Quercetin
Liposome
Arntl/Bmal1
Clock
adaption
Per3
Tim
resetting
C
EMT-3D Spheroids / ml
Day-0 Modification
Day-7 Modification
Day-14 Modification
Day 0
Day 7
Day 14
Days for dynamic suspension culture
D
3D-ETSB^Tim+
3D-ETSB^Tim-
Oct-4
Nanog
E

Fig. S6. Blockade of Clock/Arntl-Per3/Tim dynamic regulatory loop in 3D-ETSB. Related to Fig.6.

A, Immunofluorescence molecule scan was utilized to detect timeless expression in homeostatic ETSB and Arntl/Tim-low/negative ETSB (3D-ETSB^{Tim-}).

B, Since it is hard for Arntl/Tim-null OSEs (OSEs^{Tim-}) to regenerate EMT-3D-spheroids, liposomal quercetin (Lipo-Que) modification for dynamic blockade of Clock/Arntl-Tim feedback regulatory loop was adopted for the 3D-ETSB^{Tim-} preparation. About 250 3D-floating ETSB spheroids per ml were exposed to 15μg of Lipo-Que for 12 hours.

C, Lipo-Que modification at different time-points was adopted and selected for the blockade of Clock/Arntl-Tim regulatory loop and meanwhile without hindering 3D-EMT spheroid transformation from OSEs.

D, Immunofluorescence assay was used to see if the expression of Nanog (FITC) and Oct-4 (AMCA-labelled) in 3D-ETSB (same field) would be impeded synchronously by Lipo-Que modification at day 14.

E, Weighted gene co-expression network analysis (WGCNA) was performed for different gene modules based on whole transcriptome RNAseq with R-Package/Cytoscape Series and indicated that homeostatic ETSB (upper modules) would resettle Clock/Arntl-Per3/Tim-LTβR-NF-κB-RelA/B feedback loops as cascades to re-prime secondary central/thymus rhythm-peripheral/defense networks (lower modules) into innovative dynamic collaboration yet could be impeded functionally by relevant blockade.

Fig.S7

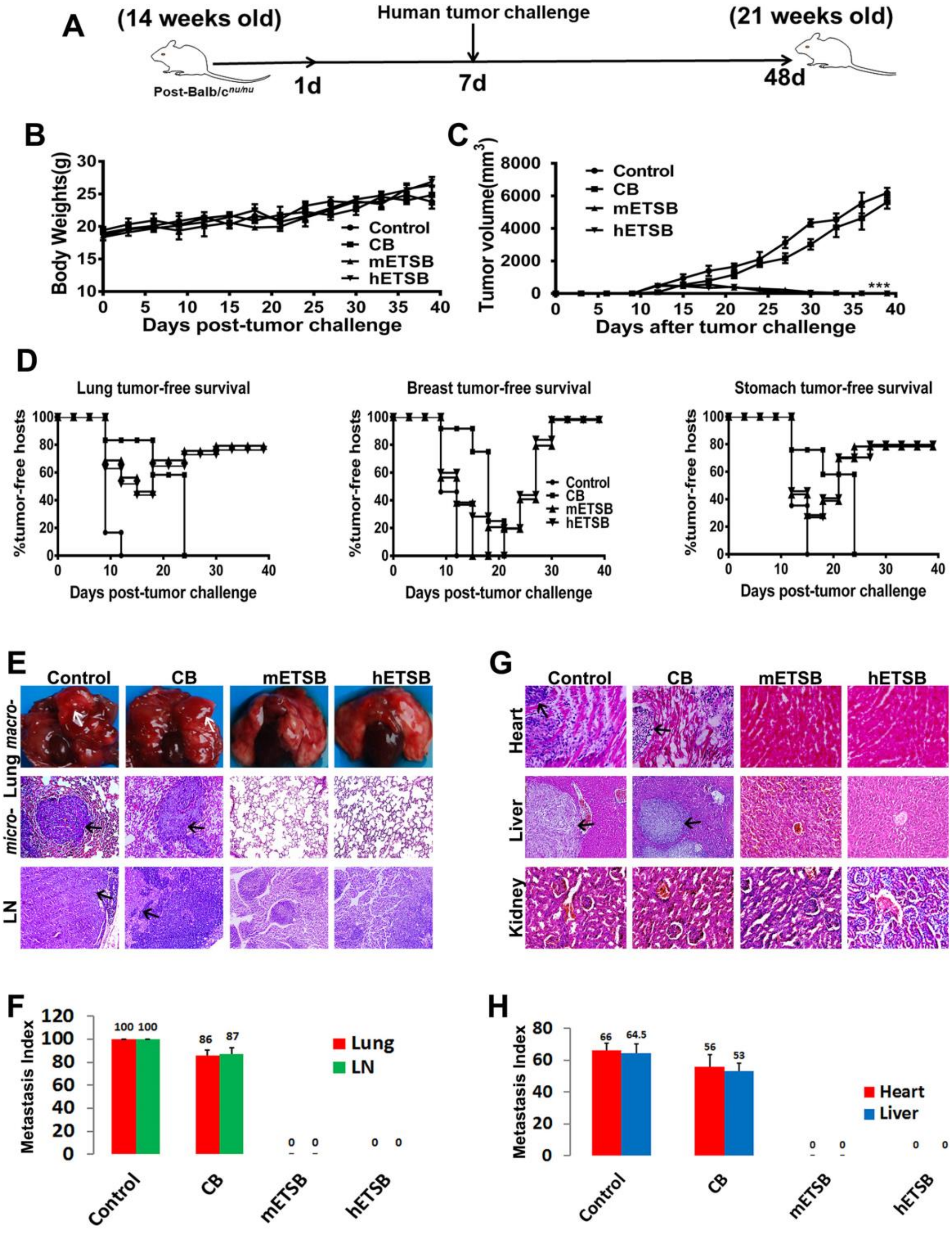

A
(14 weeks old)
Human tumor challenge
(21 weeks old)
Post-Balb/c$^{nu/nu}$
1d
7d
48d
B
Body Weights(g)
Days post-tumor challenge
Control
CB
mETSB
hETSB
C
Tumor volume(mm^3)
Days after tumor challenge
Control
CB
mETSB
hETSB
***
D
Lung tumor-free survival
Breast tumor-free survival
Stomach tumor-free survival
%tumor-free hosts
Days post-tumor challenge
Control
CB
mETSB
hETSB
E
Control
CB
mETSB
hETSB
Lung macro-
micro-
LN
G
Control
CB
mETSB
hETSB
Heart
Liver
Kidney
F
Metastasis Index
100 100
86 87
0 0
0 0
Lung
LN
Control
CB
mETSB
hETSB
H
Metastasis Index
66 64.5
56 53
0 0
0 0
Heart
Liver
Control
CB
mETSB
hETSB

Fig.S7. ETSB-innovated attack-defense dynamics deters peripheral biologic burden/tumor metastases. Related to Fig.7.

A, Experimental layout of tumor challenge to CB- and m/hETSB- inoculated hosts (Post-Balb/c$^{nu/nu}$).

B, Graph represents the body weight curves of different groups (P>0.05).

C, Graph depicts the tumorigenesis dynamics in different groups based on tumor volumes at indicated days after tumor challenge (***P<0.005).

D, Graphs represent the dynamic progression of different tumor burdens versus tumor-free survival rate at indicated days after tumor challenge (P<0.01, n=10).

E, Neoplastic metastases into lungs (***Top-Middle panels***) and draining sentinel LNs (***Lower panel***) were monitored with ***arrows*** indicating metastatic foci and ***white arrows*** indicating visible metastases, especially, with no microscopic toxic or therapeutic cell-deposited clinical or pathologic indications in pulmonary alveoli and LN tissue. Data show parts of representative results from three independent experiments with 10 mice per group.

F, As in (E), histomorphometry depicts the percent metastasis index into lungs and LNs among different groups (P<0.005).

G, 200X magnification manifests metastasis establishments in hearts and livers, with ***arrows*** indicating the foci among different groups. Meanwhile, no systemic toxicity or cell-deposited pathologic lesions, including macro-/micro-scopic indications, is detected in heart, liver or kidney from ETSB inoculation through the experiment till study termination.

H, Percent metastasis index into heart/liver among different groups has been analyzed by histomorphometry (P<0.005).

Fig.S8

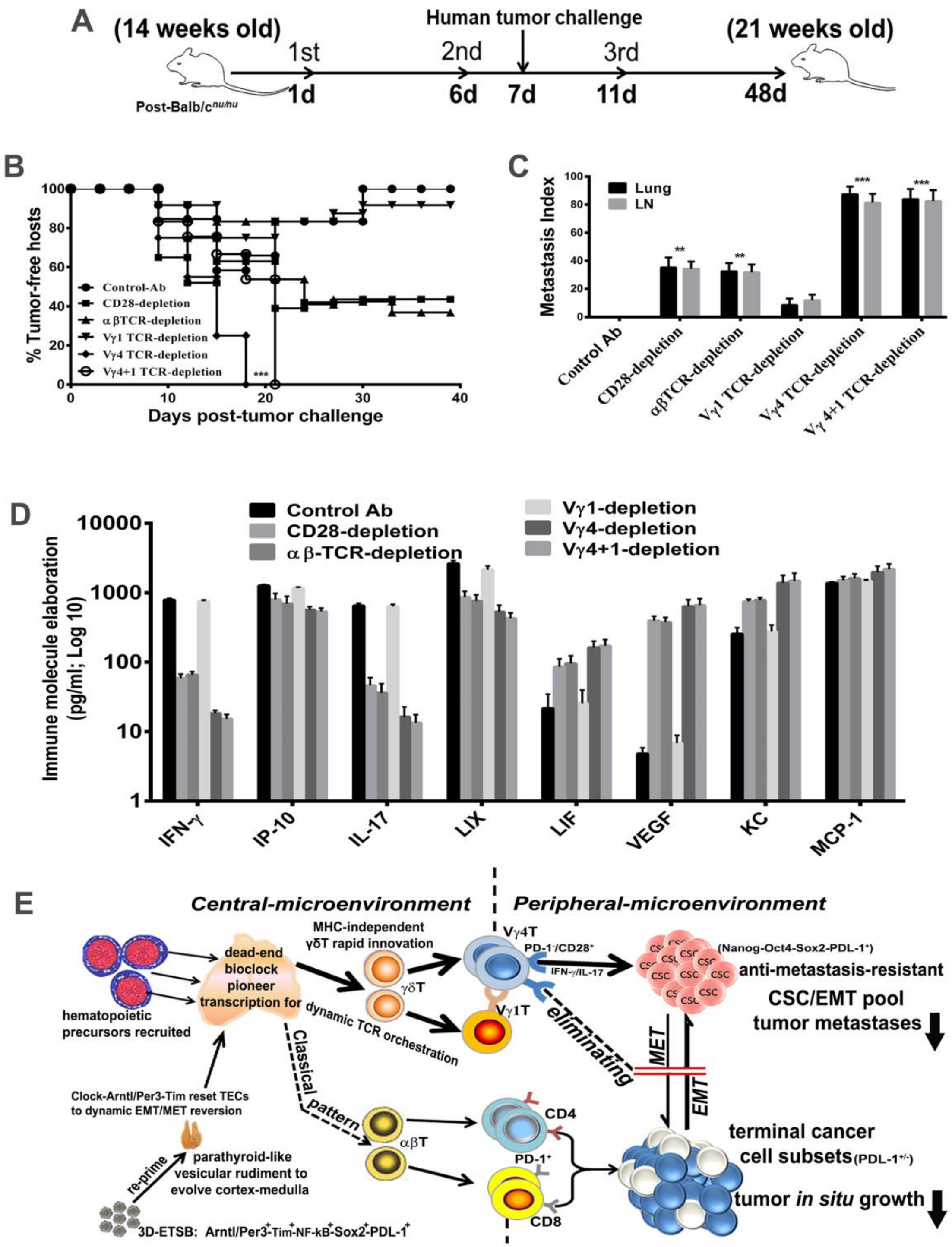
A
(14 weeks old)
Human tumor challenge
(21 weeks old)
1st
2nd
3rd
1d
6d
7d
11d
48d
Post-Balb/c nu/hu
B
% Tumor-free hosts
Days post-tumor challenge
Control-Ab
CD28-depletion
αβTCR-depletion
Vγ1 TCR-depletion
Vγ4 TCR-depletion
Vγ4+1 TCR-depletion
***
C
Metastasis Index
Lung
LN
Control Ab
CD28-depletion
αβTCR-depletion
Vγ1 TCR-depletion
Vγ4 TCR-depletion
Vγ4+1 TCR-depletion
D
Immune molecule elaboration (pg/ml; Log 10)
Control Ab
CD28-depletion
αβ-TCR-depletion
Vγ1-depletion
Vγ4-depletion
Vγ4+1-depletion
IFN-γ
IP-10
IL-17
LIX
LIF
VEGF
KC
MCP-1
E
Central-microenvironment
Peripheral-microenvironment
hematopoietic precursors recruited
dead-end bioclock pioneer transcription for
MHC-independent γδT rapid innovation
dynamic TCR orchestration
Classical pattern
γδT
αβT
Vγ4T
Vγ1T
PD-1-/CD28+
IFN-γ/IL-17
eliminating
(Nanog-Oct4-Sox2-PDL-1+)
anti-metastasis-resistant
CSC/EMT pool
tumor metastases
MET
EMT
CD4
PD-1+
CD8
terminal cancer cell subsets(PDL-1+/-)
tumor in situ growth
Clock-Arntl/Per3-Tim reset TECs to dynamic EMT/MET reversion
parathyroid-like vesicular rudiment to evolve cortex-medulla
re-prime
3D-ETSB: Arntl/Per3+-Tim+-NF-kB+-Sox2+-PDL-1+

Fig.S8. Innovated-TCR blockage impedes ETSB-primed antimetastases immunocompetence

A, The layout for relevant TCR blockage at indicated three times shown as an overview.

B, As in (A), additional hETSB-inoculated hosts (Post-BALB/$c^{nu/nu}$) were established as described above and injected with different neutralizing-mAb. Graph depicts whether tumor-free induction by ETSB could be evidently terminated by relevant TCR blockade or not **(*****P<0.005 versus Control Ab group; n=5**).**

C, Graph represents whether metastasis-free induction by ETSB could be evidently terminated by different TCR depletion or not **(****P<0.01; *****P<0.005 versus Control Ab group; n=5).

D, Magnetic bead microarray was utilized to monitor immunoregulatory microenvironment of homeostatic ETSB-inoculated hosts (Post-BALB/$c^{nu/nu}$) after TCR blockade. The regulated molecules cover IFN-γ, IP-10, IL-17, LIX/CXCL5, LIF, VEGF, KC, MCP-1/CCL2. Values expressed as means±SD as Log10; P<0.05, n=5.

E, Consequently, ETSB could re-prime molecule clock to evoke withered thymic rudiment into endogenous innovation, as Clock-Arntl/Per3-Tim axis resets TECs by undergoing dynamic MET/EMT reversion in central microenvironment for thymic progenitors remodeled and subjected to multifunctional revival of dynamic TCR orchestration to innovate innate-adaptive immunocompetence against evolving biologic burdens/tumor challenges. Where αβTCR-dominated evolving-repertoire could eliminate non-stem terminal cancer-cell subsets; Vγ4TCR-dominated evolving-subsets can accurately attack peripheral EMT/CSC-evolving pools, and are thereby able to fully address therapy-resistance/relapse-metastasis.

Supplemental Methods

In vitro EMT-3D-floating system establishment and ETSB preparation

Human ovary tissues were obtained after informed consent and ovarian surface epithelium (OSE) was digested by collagenase II for 30 min at 37°C to separate surface epithelial cells. Mononuclear cells were collected and filtered through a 70-mum nylon mesh (BD Biosciences) to weed out unwanted cells. Then the selected cells were suspended in DMEM supplemented with 15% FBS, 10U bFGF, 2 mM L-glutamine and 0.1mg Amikacin/ml at an initial seeding density of 5×10^5 cells/ml. Two days later, the nonadhesive cells were removed by washing with serum-free DMEM. Next, media were replaced twice per week for a consecutive 14-day anchorage-independent screening period in serum-free medium [31, 32] to generate floating EMT-3D spheroids via ameliorative dynamic shaking suspension model. Immunofluorescence with flow cytometry was used to detect characteristic markers of EMT-3D-spheroids for CD44, CD73, CD133, CD200, Nanog, Oct4, Sox2, PDL1, Arntl-Clock/Per3-Tim(E-15, H-276, G4). Purified EMT-3D-spheroids with positive markers were propagated in dynamic suspension with ameliorative DMEM/F12/1640-integrated medium until generating more than 250 floating 3D-spheroids/ml with 195±25μm/each D (**Fig.S1 and Video.1-2**). These spheroids were collected and X-ray ameliorated using RS-2000 biological irradiator (www.radsource.com) at 160 Gy so as to keep the cells metabolically alive yet unable to proliferate and address renewal, frozen in one batch and resuscitated as EMT-3D-spheroid biologics (ETSB). Via the same procedure, mOSE-derived ETSB (mETSB) was prepared from C57BL/6 mice ovary by the same formula. Common OSEs in adherent culture were X-ray-ameliorated as common 2D-biologics (CB). High-throughput transcriptome sequencing and quantitative RT-PCR were collectively used to detect dynamic transcriptional characteristics of EMT-3D-spheroid establishment.

qRT-PCR analysis

RNA was isolated using an RNeasy Mini Kit (Qiagen) and subjected to on-column DNase digestion. Less than 5 mg total RNA was reverse transcribed to cDNA with 1st strand cDNA synthsis (PrimeScript^{TM}RT reagent Kit with gDNA Eraser, Takara); qPCR amplification for certain primers were performed with SYBR Premix Ex Taq^{TM} II (Takara) and CFX96 Real-Time PCR Detection System (Bio-Rad). The relative

expression of mRNA was normalized to β-actin expression and calculated by using the $2^{-\Delta\Delta CT}$ method. All primers were designed with NCBI Primer Blast, primer sequences for amplification were listed in Supplemental Table S1-S4. Data from qRT-PCR were analyzed with GraphPad Prism Version 6.0, differences between groups were statistically evaluated by sample one-tailed Student's t-test with $p<0.05$ considered as significant. Genomic DNA was extracted and purified from the rhesus PBMCs using TIANamp Genomic DNA kit (TIANGEN). The DNA concentration was then determined using NanoDrop2000. qRT-PCR assays for rhesus TREC quantification[33, 34] were performed in quadruplicate and TREC number per genomic DNA (100 ng) was calculated using the software provided with the Bio-Rad CFX96 Real-Time System and LightCycler 480 System (Roche Diagnostics).

Inoculation regimen in immune deficiency and senescence hosts

Research protocol involving animals was reviewed and approved by institute's Animal Care and Use Committee. Athymic Balb/$c^{nu/nu}$ nude mice 6 weeks of age, C57BL/6 and Balb/c mice with the same weeks were purchased from Huafukang Bioscience Company and maintained in air-filtered laminar flow cabinets under aseptic conditions with a 12-h light/dark cycle. *Arntl/Bmal1*$^{-/-}$ knockout model was constructed by Cyagen through the deletion of Exon 6,7,8 and 9 in arntl gene locus of chromosome 7 on C57BL/6 backgrounds using CRISPR/Cas9 techniques. Mice were fed with AIN-93M rodent diet and autoclaved reverse-osmosis treated water to ensure proper health and acclimate to their living environments before study initiation. One week later the mice were randomly assigned to Control, CB and mETSB and hETSB groups with 24 hosts in each group including 12 females and 12 males. ETSB group hosts were subjected to four times of ETSB of subcutaneous inoculation into left flank (5×10^3 of biologics spheroids per time). Primary inoculation was followed by the second inoculation 2 weeks later, then by 1 week apart. Hosts after receiving four times of subcutaneous ETSB-inoculation would be used as Post-Balb/$c^{nu/nu}$ for further study. Control group and CB groups were subjected to corresponding inoculation regimens. Targeted irradiation for normal mice at week 6~7 was performed in a specialized chamber exposing only the thymic area to a targeted dose (9.5Gy) of X-ray radiation. Adult rhesus macaques 5-6-year old, have lived their entire lives at the Primate Research Center of Scientific Park, and have known birthdates, pedigrees, and complete medical histories.

Prior to the start of this study, no animals had any clinical or experimental history that would be expected to affect disease susceptibility or immunity. The animals have been fed a semi-purified, nutritionally fortified, low fat diet containing 15% protein and 10% fat and lived under the circadian model condition (Natural light regime, 16 h-light: 8 h-dark cycle, LD 16:8, with ZT0 defined as lights on) with drinking water and daily activities freely. As part of the study design, animals were evenly matched and randomized to Control, CB or (h)ETSB groups with 6 hosts in each group including 3 females and 3 males and treated for presenting conditions. The hosts received five times of (h)ETSB inoculations (5×10^5 of biologics spheroids per time) subcutaneously into both upper arms, or other corresponding regimens during two years (*Month 0-1-6-12-24* protocol), with subsequent relevant detections covering thorax MRI two year more later. Every subject was sampled for the peripheral blood 5ml every 4 hours at each circadian time point (ZT2, ZT6, ZT10, ZT14, ZT18 and ZT22) in a circadian day. Lymphocytes were separated from blood and total RNA was extracted from each sample. RT-PCR was used to determine the temporal changes in mRNA levels of Clock/Arntl and other core genes during different zeitgeber times. The circadian parameters were obtained and analyzed by both cosine function/Cosinor analyses and amplitude *F* test to reveal the rhythmic transcriptions of core clock genes in LD (16:8) condition.

Integral detection for endogenous immune-innovation dynamics

Revival dynamics of central/peripheral integral microenvironments for Pre-/Post- Balb/$c^{nu/nu}$ hosts with relevant organs and tissues were *in vivo* detected using MRI dynamic molecule scanning system (Bruker Bio-Spec 70/30USR) and PerkinElmer IVIS Lumina Series III (PerkinElmer Inc.), with Leica confocal molecule scanning system used to monitor cytokeratin 5(EP160IY), cytokeratin 8(C51), Clock/Per3-Tim (E-15, H-276, G4), TCRαβ(H57-597), TCRγδ(GL3), TCR-Vγ1(clone 2.11) or -Vγ4(UC3) for relevant molecules and intravital TCR revival levels via 488, 568 and 633nm filters; then the TCR repertoire innovation was further analyzed via FACS-Aria Ⅲ cell sorting system.

High-throughput transcriptome-sequencing and ELISpot assay with thymic rudiment nursing

Whole transcriptome RNAseq library was prepared for dynamic transcriptional characteristics detection of thymocytes and splenocytes. Total RNA was isolated from tissues using a Trizol reagent (ambion, life

technology). The RNA quality was assessed using a BioAnalyzer 2100 (Agilent, Santa Clara, CA, USA), and the samples were stored at −80°C until use. RNA integrity numbers (RIN) of these RNA samples have succeeded 8.0 and were appropriate for cDNA library construction using TruSeqTM RNA Sample Preparation Kit. The libraries were established by NEBNext Ultra RNA Library Prep Kit for Illumina, purified by Beckman AMPure XP beads, and quantified by ABI 7500 real time PCR system using KAPA SYBR green fast universal (2x) qPCR master mix. Samples were clustered on cBot cluster generation system and sequenced on HiSeqTM 4000 platform according to manufacturer's instructions. The Dual-Color IFN-γ/IL-4/17 ELISpot Kits (R&D Systems# ELD5217) was adopted for relevant cells monitoring. Briefly, thymocytes were harvested from thymus of Post-Balb/$c^{nu/nu}$ hosts. 5×10^4 recipient thymocytes as responder cells and 3×10^3 ETSB cells as biological burden/stimulators were respectively performed according to manufacturer's protocol. 72 hours later, spots were automatically scanned using an ELISpot plate reader (Cellular Technology Ltd., Cleveland, OH) for scanning. Vesicular thymic rudiments were harvested from Balb/$c^{nu/nu}$ nude mice 7 weeks of age and nursed in RPMI 1640 supplemented with 20% FBS at 37°C with 5% CO_2 for one week; and then nursed in ameliorative DMEM/F12/1640 integrated medium with 3D-ETSB at 120 spheroids/ml for another four weeks under floating separation condition via 6.5-μm filter screen for avoiding direct attachment between thymic rudiments and ETSB.

Side effect evaluation

Health status of all inoculated hosts was observed successively for relevant clinical indexes such as weight loss, ruffled fur, diarrhea, anorexia, cachexia, skin ulceration or toxic deaths. The tissues of heart, liver, spleen, lung, kidney, brain, and so on were fixed in 4% neutral buffered formalin solution and embedded in paraffin. Slices of 4 microns were stained with hematoxylin and eosin (HE) and observed by two pathologists in a blinded manner.

TCR revival monitor and blockade reactivity to tumor challenge

MDA-MB-231 mammary, SPC-A1 lung and MGC803 stomach tumor cell lines were obtained from American Type Culture Collection (ATCC, Rockville, MD) and propagated by in vitro passage in DMEM (Gibco BRL, Grand Island, N.Y) with 10% of FBS (Gibco, Auckland, N.Z). Post-Balb/$c^{nu/nu}$ hosts 14~15 weeks of age were

challenged with 5×10^5 MDA-MB-231, SPC-A1 or MGC803 tumor cells subcutaneously into right flank. Tumor growth and metastasis, covering detection of the inguinal sentinel LNs, were monitored once per three days consecutively by measuring the largest diameter and respective perpendicular diameters using a caliper and plotted at three-day intervals throughout the whole experiment. Tumor volume (mm^3) was determined by the formula: $0.52\times$length (mm)$\times$width^2(mm)^2. As for TCR pathway blockade experiments, additional post-Balb/c$^{nu/nu}$ hosts were established as described above and performed by i.v. injection of anti-Vγ1TCR (clone 2.11, 100 mg/mouse), anti-Vγ4TCR (clone UC3, 100 mg/mouse), anti-CD28 (27.51 mAb) or anti-αβTCR (clone H57-597, 100 mg/mouse) mAb on 7 days and 1 day before and 4 days after tumor-challenge, with normal rat IgG injected according to the same protocol as control. Depletion was confirmed by FACS analysis, and then hosts were allowed for a 6-week observation period for tumor growth and metastasis.

Arntl-Tim-low/negative ETSB preparation by liposomal quercetin modification.

Liposomal quercetin (Lipo-Que) was prepared using a solid dispersion method as previously described[35, 36]. Briefly, the mixture of lecithin, cholesterol, PEG, and Que was dissolved in chloroform/methanol (3:1, v/v), and evaporated to dryness using a rotary evaporator under reduced pressure. The lipid films were dissolved in 5% glucose solution under ultrasonication, and subsequently concentrated and lyophilized. About 250 3D-floating ETSB spheroids per ml at day-14 were exposed to 15μg of Lipo-Que for 12 hours[37, 38]. Control cultures as homeostatic ETSB were left untreated. Clock/Per3-Tim blockades were detected by immunofluorescence for the ETSB and then evaluated using qRT-PCR analysis.

Magnetic bead microarray and western blotting

Non-necrotic fresh tumors or tumor-free local inoculum and thymus were cut into small pieces, homogenized in liquid nitrogen, emulsified by ultrasonication, extracted with ice-cold RIPA lysis buffer at a ratio of 100mg:1ml, cracked 30min onto ice, passed through a fine mesh sieve (Bellco Glass) and then centrifuged (12000rpm, 20min, 4°C). The extracted total protein was concentrated to 12mg/ml and then detected for peripheral molecule microenvironment of immunoregulatory network according to MILLIPLEX® MAP magnetic bead panel kit (Luminex, USA). Nanog and IL17 levels were determined by the blotting using 50μg total protein from each sample.

Statistical analysis

SPSS software package system was used for most statistical analyses. Data were subjected to one-way ANOVA plus Tukey post-hoc test or two-way ANOVA and repeated measures when comparing more than two groups. Log-rank test and Kaplan-Meier method were used to analyze tumor-free survival differences. Numerical values are reported as means ± one standard deviation (SD). Statistical significance was assumed for $P<0.05$. Weighted gene co-expression network analysis (WGCNA) in bioinformatics was performed for different gene modules with R Package (version 3.2) as well as Cytoscape (version 3.6.0) based on whole transcriptome RNAseq. Heatmaps were generated by R package with hierarchical clustering algorithm .

Antibodies used for IF and Flow cytometry

**Antibody**	**Clone**	**Supplier**	**Identifier**
Alexa Fluor R647 Mouse anti-Oct3/4	40/Oct-3	BD Pharmingen™	560307
PE Mouse anti-Mouse Nanog	M55-312	BD Pharmingen™	560277
PE Mouse anti-human Nanog	N31-355	BD Pharmingen™	560873
Alexa Fluor R488 Anti-Human/Mouse Sox2	Btjce	eBioscience	Cat#53-9811
Vimentin Rabbit mAb Alexa Fluor R647 Conjugate	D21H3	Cell Signaling Technology	Cat#9856
FITC Mouse Anti- E-cadherin	36/E-cadherin	BD Pharmingen™	612131
PE-Cy™7 Rat Anti-Mouse CD44	IM7	BD Pharmingen™	560569
PE Mouse Anti-Human CD44	G44-26 (also known as C26)	BD Pharmingen™	561858
Alexa Fluor® 488 Rat Anti-Mouse CD73	TY/23	BD Pharmingen™	561545
FITC Mouse Anti-Human CD73	AD2	BD Pharmingen™	561254
PE-Cy™7 Rat Anti-Mouse CD90.2	53-2.1	BD Pharmingen™	561642
PE Mouse Anti-Human CD90	5E10	BD Pharmingen™	555596
APC anti-mouse CD200 (OX2)	OX-90	Biolegend	Cat#123810
PE-Cy™7 Mouse Anti-Human CD200	MRC OX-104	BD Pharmingen™	562125
Mouse PD-L1/B7-H1 Antibody	929903	R&D Systems	MAB9078
PD-L1 Rabbit mAb (Alexa Fluor® 488 Conjugate)	(E1L3N®) XP®	Cell Signaling Technology	#14772
CD133/1-APC, human	AC133	Miltenyi Biotec	130-090-826

Anti-Prominin-1-APC, mouse	CD133	Miltenyi Biotec	130-092-335
CD133/1 pure	AC133	Miltenyi Biotec	130-090-422
αβTCR-FITC	H57-597	Abcam	Ab25010
PE Hamster Anti-Mouse γδ T-cell Receptor	GL3	BD Pharmingen™	553178
FITC Hamster Anti-Mouse γδ T-cell Receptor	GL3	BD Pharmingen™	561996
PE anti-mouse TCR Vγ1.1/Cr4 Antibody	2.11	BioLegend	Cat#141106
Purified anti-mouse TCR Vγ1.1/Cr4 Antibody	2.11	BioLegend	Cat#141102
FITC Hamster Anti-Mouse Vγ 4 T-cell Receptor	UC3-10A6	BD Pharmingen™	553226
Anti-Cytokeratin 5 antibody	EP160IY	Abcam	Ab52635
Cytokeratin 8-FITC	C51	Santa Cruz	sc-8020FITC
FITC Rat anti-mouse CD4	RM4-5	BD Pharmingen™	553046
PE Rat Anti-Mouse CD8a	53-6.7	BD Pharmingen™	553032
Purified anti-mouse CD4	GK1.5	Biolegend	Cat#100402
Purified anti-mouse CD8a	53-6.7	Biolegend	Cat#100702
PerCP Hamster Anti-Mouse CD3e	145-2C11	BD Pharmingen™	553067
Purified NA/LE Hamster Anti-Mouse CD3e	145-2C11	BD Pharmingen™	553057
4',6-Diamidino-2-Phenylindole,Dihydrochloride (DAPI)		Invitrogen™	D1306
Anti-GAPDH antibody	D16H11	Cell Signaling Technology	Cat#5174
In Vivo MAb anti-mouse TCR Vγ1.1/Cr4	2.11	Bioxcell	Cat#BE0257

In Vivo MAb Vγ4TCR	UC3-10A6	Bioxcell	Cat#BE0168
Purified NA/LE Hamster-anti-Mouse CD28	37.51	BD Pharmingen™	553294
Purified NA/LE Hamster-anti-Mouse TCR β-Chain	H57-597	BD Pharmingen™	553166
Purified NA/LE Hamster-anti-Mouse γδ T-cell Receptor	UC7-13D5	BD Pharmingen™	553181
Per3	E-15	Santa Cruz	sc-12017
Clock	H-276	Santa Cruz	sc-25361
Timeless	G-4	Santa Cruz	sc-393146
BMAL1 (Arntl)	A6	Santa Cruz	sc-373955
APC Mouse Anti-Human CD3	UCHT1	BD Pharmingen™	561810
PE-CF594 Mouse Anti-HumanCD45RA	5H9	BD Pharmingen™	565419
FITC Mouse Anti-Human CD45RA	HI100	BD Pharmingen™	561882
FITC Mouse Anti-Human CD31	WM59	BD Pharmingen™	557508
APC-R700 Mouse Anti-Human CD27	M-T271	BD Pharmingen™	565116
IL-17	G-4	Santa Cruz	sc-374218
Nanog	H-2	Santa Cruz	sc-374103
Sox2		Cell Signaling Technology	#2748
Oct-4	V241	Cell Signaling Technology	#2788

Table S1. Primers used for human qRT-PCR

Primers		Sequences（5'-3'）
β-actin	forward	CCACGAAACTACCTTCAACTCC
	reverse	GTGATCTCCTTCTGCATCCTGT
Oct4	forward	GGAATCTGGACCTGAGCGAG
	reverse	ACTGACTTCCGGAACGAACC
Sox4	forward	GCACTAGGACGTCTGCCTTT
	reverse	ACACGGCATATTGCACAGGA
Snail	forward	CCAGTGCCTCGACCACTATG
	reverse	CTGCTGGAAGGTAAACTCTGGA
NF-κB	forward	AGTCCCTCTGTTCCCGAAGG
	reverse	CCGGGTAATGGGGAAGAAGG
Twist1	forward	TCTCGGTCTGGAGGATGGAG
	reverse	TCTCTGGAAACAATGACATCTAGG
Arntl	forward	GCTTCTGCACAATCCACAGC
	reverse	TCGTTGTCTTCATCCAGCCC
Stat3	forward	TCCTGAAGCTGACCCAGGTA
	reverse	TATTGCTGCAGGTCGTTGGT
Clock	forward	TGGAGCGAGAGCGCGAA
	reverse	CAGCAGCTTTGCAGGAACAA
Timeless	forward	GAACGGCAGGAGGAAGACAA
	reverse	CCTGATCAAGGTCAGCTGGG
Per3	forward	AACTGGACCCAAAGAGCAGC
	reverse	GATCCGGAATGGGGAGTGAC

Table S2. Primers used for mouse qRT-PCR

Primers		Sequences(5'-3')
β-actin	forward	CCTCTATGCCAACACAGTGC
	reverse	ACATCTGCTGGAAGGTGGAC
LTβR	forward	CCAGATGTGAGATCCAGGGC
	reverse	CCTGGCTCTGGGGGATTTTT
NF-κB	forward	GTAGACAGCCCCATTCAGGG
	reverse	AGAGGGAAGAAGCCACCAGA
Bcl-xL	forward	CCTCCTCCCCGACCTATGAT
	reverse	CAAATCTATCTCCGGCGACCA
Icam	forward	TCCGCTGTGCTTTGAGAACT
	reverse	TCCGGAAACGAATACACGGT
Traf	forward	AGCTACTTCCGGTTGCAGTC
	reverse	ATGCCAGCTAGTGGAGGGTT
Tlr4	forward	TCTGGGGAGGCACATCTTCT
	reverse	AGGTCCAAGTTGCCGTTTCT
Nanog	forward	CCTCGCCATCACACTGACAT
	reverse	GCGTTCCCAGAATTCGATGC
RelA	forward	CTGTGTACCGTCATCCAGGG
	reverse	AGTGGAAGCCCTGTCCTAGT
RelB	forward	TAGGGGCCTTGGGTTCCAGT
	reverse	TTCCAATTCATCTGTGGTCCTGG
K5	forward	TGTTGCACGTCAGCACCTAT
	reverse	AGGGATGGGGTTCTGCTTTG
K8	forward	TATGGGGGACTCACTAGCCC
	reverse	TGAAAGTGTTGGATCCCCCG
Clock	forward	TTGTCAGCACTAGCTGCCTC
	reverse	ATACTGGAGTCGTGTGGGGA
Timeless	forward	CCACTGGGGAGGAAGAATGG
	reverse	TTGCCTCCTCCCCTCATCAT
Per3	forward	CCGCCCCTACAGTCAGAAAG
	reverse	TGAGCCCCACGTGCTTAAAT
Arntl	forward	CTGCCCTCTGGAGAAGGTG
	reverse	TTCCTCCGCGATCATTCGAC

Table S3. Primers used for mouse thymus MHC identification qRT-PCR

Primers		Sequences（5'-3'）
Mus β-actin	forward	CCTCTATGCCAACACAGTGC
	reverse	ACATCTGCTGGAAGGTGGAC
MHC	forward	CCTCACCCTGAGATGGGGTAA
	reverse	CTCAGGGTCTCCAGAAGGCTC
HLA	forward	TCTTGGAGTACTCTACGGGTGA
	reverse	TATCTGTCCAGGAACCGCAC

Table S4. Primers used for Macaca Rhesus qRT-PCR

Primers		Sequences（5'-3'）
TREC	forward	ATCACTCTGTGTCTAGCTCCCAGC
	reverse	ACTTGCTGAGTTTCATGATTCCTCTA
GAPDH	forward	GAAATCCCATCACCATCTTCCAGG
	reverse	GAGCCCCAGCCTTCTCCATG
Clock	forward	CAGTTGGAACAACGGACACG
	reverse	TGCAAAAACATCTGCAGCCC
Arntl	forward	TATCGCCAAAGTCAAGGAGC
	reverse	CGTCGTGCTCCAGAACATAA
CRY1	forward	AGCTCCATGGGTACTGGTCT
	reverse	AGTATTCCTCCTGAACGTTTTCTAA
Per3	forward	GCTGAGTATCAGTGTGTTGCAG
	reverse	ATGGACAGTGCACCGGTAGT
Timeless	forward	GGGGGCTCGTATATTGTCCA
	reverse	ATCCCATGAGCCGGTTGTAA
NF-κB	forward	TTGGGAAGGCCTGAACAAATG
	reverse	AGATATGGGCCATCTGCTGTT
LTβR	forward	GTGCCACATGTGCCGAAAAT
	reverse	CCCATCACTGGGTCACAGG
RelA	forward	TCCCGTGCGTGTCTCCAT
	reverse	CCATCCCAGCTGTGTACCTG
RelB	forward	CTCGCGACCATGACAGCTAC
	reverse	TTTTCTTCCGCCGTTTGCTC

Table S5. Cosinor analyses for mRNA rhythmic transcriptions of bHLH-Clock genes in rhesus macaques of different groups under LD (16:8) condition.

Group	genename	Period	Robustness	Mesor	Amplitude	Acrophase	*p*
Control	Clock	26.0 h	50.50%	1.3578	0.3373	-251°	0.005383
	ARNTL	26.0 h	70.20%	1.3824	0.5232	-254°	0.000246
	CRY1	20.0 h	75.10%	1.6587	0.6095	-199°	0.0001
	Per3	21.6 h	64.50%	1.1887	0.5851	-300°	0.000646
	Timeless	26.0 h	73.10%	0.623	0.4109	-5°	0.000147
	LTbR	21.9 h	16.40%	1.1951	0.1377	-291°	0.261101
	NF-kB	20.4 h	6.80%	1.1955	0.1346	-143°	0.602328
	RelA	26.0 h	29.30%	1.2738	0.1839	-233°	0.073074
	RelB	25.0 h	28.50%	1.1523	0.1692	-265°	0.07917
	TREC	26.0 h	48.10%	1.5015	0.4166	-214°	0.007432
CB	Clock	22.6 h	70.40%	1.8445	0.799	-259°	0.000238
	ARNTL	26.0 h	77.80%	2.2966	1.0431	-264°	0.000059
	CRY1	20.0 h	68.80%	1.7342	0.3458	-149°	0.000315
	Per3	23.0 h	76.10%	1.4176	0.9905	-264°	0.000083
	Timeless	25.2 h	58.50%	1.2272	0.6479	-29°	0.001666
	LTbR	26.0 h	20.80%	2.0857	0.3173	-319°	0.172808
	NF-kB	20.0 h	12.20%	2.5565	0.1857	-355°	0.378214
	RelA	26.0 h	9.70%	1.4676	0.1269	-212°	0.530665
	RelB	26.0 h	15.20%	1.4868	0.1724	-295°	0.290609
	TREC	20.0 h	43.60%	3.6391	0.4766	-263°	0.013525
hETSB	Clock	20.0 h	0.755	3.902	0.915	-7°	0.000093
	ARNTL	20.0 h	0.479	4.5317	0.9735	-28°	0.007603
	CRY1	26.0 h	0.668	5.4147	1.3685	-172°	0.000441
	Per3	20.1 h	0.882	5.6475	2.0433	-325°	0.000005
	Timeless	20.0 h	0.775	5.4893	1.1373	-1°	0.000063
	LTbR	21.5 h	0.928	6.5579	2.242	-132°	0.000001
	NF-kB	23.1 h	0.33	8.313	1.1306	-256°	0.048727
	RelA	21.1 h	0.664	4.5677	1.1117	-257°	0.000473
	RelB	24.5 h	0.775	7.2262	1.3598	-147°	0.000063
	TREC	20.9 h	0.842	12.7706	5.1298	-290°	0.000015

Video 1: Establishment of EMT-3D floating system. Related to Fig.1.

OSEs remain un-anchorage by dynamic shaking suspension to regenerate tridimensional (3D)-EMT spheroid transition in ameliorative DMEM/F12/1640-integrated medium. More than 250 floating EMT-3D-spheroids could be enriched per ml for about 15 days of ameliorative dynamic suspension, with an efficacy of mesenchymal-transition over 80%. Routine cultures could not generate 3D-spheroid transition.

Video 2: Phenotype of EMT-3D floating-spheroid. Related to Fig.1/S1.

OSEs are propagated by dynamic shaking suspension system to regenerate3D-EMT spheroid pluripotent conversation in ameliorative DMEM/F12/1640-integrated medium. Each spheroid contains more than 320 double-positive (CD200/CD133) EMT cells with about 195±25μm /each D for synchronous multiepitope-integration.

Video 3: Multidimensional dynamic scaling for EMT-3D-spheroid. Related to Fig.1H.

Whole transcriptomes were subjected to MDS on expressed genes to assess sample diversity and relatedness among 3D-ETSB (green), 2D-CB (red) and Control/wild OSEs (black). ETSB symbols clustered more together than CB, meaning closer relatedness within ETSB samples. Meanwhile, ETSB samples are very distinct from CB and Control samples, indicating diverse transcriptional characteristics among them and dynamic variation of Control/wild OSEs until transcription terminating.

Video 4: Multidimensional dynamic scaling of peripheral splenocytes. Related to Fig.S4C. As subjected to MDS on expressed genes, unorchestrated splenocytes from CB (red) and Control (black) groups cluster much closely together than multifunctional ETSB (green for mETSB & blue for hETSB) able to address peripheral multiple burdens.
